# Differential Biological Effects of *Trifolium pratense* Extracts—In Vitro Studies on Breast Cancer Models

**DOI:** 10.3390/antiox13121435

**Published:** 2024-11-22

**Authors:** Lucian Albulescu, Alexandru Suciu, Mihaela Neagu, Cristiana Tanase, Sevinci Pop

**Affiliations:** 1 Biochemistry & Proteomics Laboratory, “Victor Babes” National Institute of Pathology, 050096 Bucharest, Romania; albulescu.l@gmail.com (L.A.); bioch@vbabes.ro (C.T.); 2 Research Department, SC Hofigal Export-Import SA, 042124 Bucharest, Romania; alexandru.suciu287@gmail.com (A.S.); mihaela.neagu@hofigal.eu (M.N.); 3 “Nicolae Cajal” Institute of Medical Scientific Research, “Titu Maiorescu” University, 040441 Bucharest, Romania; 4 Cell Biology Laboratory, “Victor Babes” National Institute of Pathology, 050096 Bucharest, Romania

**Keywords:** red clover, plant extracts, hormetic, biphasic dose-response effects, chemoprevention, oxidative stress, antioxidant, pro-oxidant, antiproliferative, pro-proliferative

## Abstract

The increasing popularity of herbal supplements emphasizes the need of scientific data regarding their health benefits and possible toxicological concerns. The complexity of botanical extracts, which include thousands of distinct compounds, contributes to the challenging nature of this endeavor. In this study, we explored the hormetic effects of two *Trifolium pratense* extracts on breast cell lines. Using a wide range of concentrations (0.1 to 3.33 mg/mL), we analyzed how extracts modulate cellular processes such as viability, proliferation, and oxidative stress on breast adenocarcinoma highly invasive estrogen receptor negative (ER-) and noninvasive ER+ cells, as well as on non-tumorigenic ER- normal cells. The cytotoxicity and real-time cell analysis (RTCA) assays showed that both extracts exercised a biphasic dose effect on adenocarcinoma ER+ and normal ER- cell proliferation and oxidative stress. We report a monotonic dose-dependent cytotoxicity on highly invasive adenocarcinoma ER- cells; the induced apoptosis was based on the pro-oxidant activity of extracts. The reactive oxygen species (ROS) generation by high-dose ethanolic extract was observed in all cells, followed by mitochondria dysfunction. Oxidative stress parameters, such as malondialdehyde (MDA) and reduced glutathione (GSH) levels, and superoxide dismutase (SOD) activity were affected. Our study demonstrates that *T. pratense* extracts have chemoprevention potential in normal and tumorigenic breast cells by modulating cellular proliferation and oxidative stress.

## 1. Introduction

Nowadays, the herbal supplement demand has grown worldwide with an expected market size to reach USD 205 billion by 2030 [1]. Although herbs have been used from ancient times for health promotion as well as for prevention and treatment of chronic diseases, the scientific basis of their bioactivity, safety and efficacy is still largely underinvestigated. The medicinal plants are identified as rich sources of phytochemicals, which can have potential applications in both cancer prevention and the development of new drugs. Actually, the modern medicine includes more than 80% of antimicrobial, cardiovascular, immunosuppressive, and anticancer drugs with origin from plants or other natural sources [2].

Dietary chemoprevention is based on the principle that bioactive compounds found in diets rich in vegetables, fruits, or plant-based supplements can act as preventive agents to reverse or neutralize carcinogenesis. The chemoprevention involves action in multiple cellular events, including inhibition of precancerous and cancer cell proliferation, modulation of cell cycle, induction of programmed cell death (apoptosis), suppression of inflammatory factors and enhancement of antioxidant cellular defense systems [3]. Phytochemicals have demonstrated the capacity to regulate specific cellular events by acting as antioxidant, anti-inflammatory, antiangiogenic, or immunostimulatory agents. At molecular levels, phytochemicals can modulate epigenetic alterations connected to the onset and progression of cancer [3,4,5,6].

Currently, due to the increased popularity of herbal products, the scientific community is making efforts to document and scientifically validate the pharmacological potential of plant extracts. A botanical supplement can comprise hundreds or even thousands of distinct bioactive compounds at different concentrations, so it is considerably challenging to identify a specific phytochemical or class of compounds linked to a particular biological activity of a plant extract. The overall efficacy arises from the collective action of various compounds that may exhibit synergistic, additive, or even antagonistic biological activities. Whether it is due to cumulative or complementary effects or enhanced bioavailability of compounds, in-depth in vitro and in vivo research is needed to confirm the beneficial effect of a botanical supplement to establish the limits of its toxicity and, then, translate the results to clinical trials.

The exogenous antioxidant molecules are intended to protect and balance the cellular redox homeostasis. While lower levels of reactive oxygen species (ROS) are required for cell proliferation and signal transduction, prolonged exposure to even moderate levels of ROS can compromise the cellular antioxidant defense system, favoring ROS accumulation and resulting in oxidative modifications to DNA, lipids, and proteins [7]. The oxidative damage process is recognized as one of the significant contributors to cancer development and progression [7,8]. The antioxidant properties of numerous plant-based phenolics have been thoroughly investigated, emphasizing their capacity to prevent oxidative stress and inhibit carcinogenesis [8]. However, the pro-oxidant potential of bioactive compounds found in food or dietary supplements has been relatively understudied, frequently linked to toxicity.

Recently, compelling evidence has shown that individual phytochemicals or plant extracts can have nonmonotonic responses and, depending on the type of target cells or concentration, can exert opposite effects [9,10,11]. Particularly, flavonoids and other poly or monophenolics can act as antioxidants and pro-oxidant molecules depending on the dose, cellular environments, and the type of target cells. This biphasic dose–response phenomenon induced by naturally occurring molecules is raising concerns that these molecules can cause adverse effects, acting as cancer promotors, or they can produce beneficial effects by enhancing ROS-governed cancer chemotherapy approaches [12].

Moreover, the capacity of a particular class of phytochemicals, namely, phytoestrogens, which mimic the estrogen hormone’s structure, to exert opposite effects as agonists and antagonists of estrogen receptors (ERs) has been demonstrated by in vitro and in vivo studies [13]. The phytoestrogens’ dual activities have been reflected in their biological effects in the cellular context, for example, acting to stimulate breast cancer cell proliferation or inhibit their growth depending on dose and estrogen receptor status [14]. Furthermore, the consumption of supplements or foods rich in phytoestrogens can cause adverse effects on the reproductive system in animals and humans when a defined threshold has been exceeded [15].

Currently, phytochemicals are considered hormetic compounds, which, along with drugs and other stressors, induce mild cellular responses [9,16]. The hormesis concept describes the cellular adaptive response characterized by a nonlinear relationship between different doses of a compound and their biological effects. A stimulation of a cellular process at low doses and a disruption at high doses, leading to damaging effects, were observed in many cases. Thus, a high range of phytochemicals and food supplements, such as resveratrol, curcumin, chalcones, ferulic acid, ginseng, and green tea among many others, have presented dose–response opposite effects when analyzed in the cellular context in vitro or in vivo studies [16,17]. The underlying molecular mechanisms of hormetic biphasic dose effects are not fully understood, but several hypotheses, including that hormesis is related to interactions of phytochemicals acting as ligands with two target receptors that have functionally opposite responses at different concentrations, have been proposed [17]. Moreover, even though hormesis is highly complex, few signaling pathways have been identified as the ones mediating the hormetic effects, namely, sirtuin—FOXO, nuclear factor kappa-B (NF-κB), mitogen-activated protein kinase (MAPK), adenosine 5′-monophosphate (AMP)-activated protein kinase (AMPK), and nuclear factor erythroid 2-related factor 2—antioxidant response element (Nrf-2—ARE) pathways [18]. All these signaling pathways are interconnected and play critical roles in maintaining cellular homeostasis, regulating oxidative stress, and influencing cell survival and metabolic processes.

Nevertheless, it is important to identify hormetic biphasic dose effects induced by an individual natural compound or by herb-based supplements for risk assessment and toxicological evaluations. Notably, in view of their chemopreventive potential, it is important to investigate the metabolic and oxidative stress cellular response that may prevent cancer cells from metastatic dissemination and sustain the anticancer therapy such as the pro-oxidant activity of phytochemicals and herb extracts [12].

Red clover (*Trifolium pratense*) is a perennial plant part of *Leguminosae* family and has been used for centuries as an analgesic, antioxidant, anti-inflammatory, anti-dermatosis, and expectorant remedy to ameliorate various health conditions [19]. Based on its chemical content, the plant has been identified as a valuable source of nutraceuticals and bioactive compounds, mainly with estrogenic and antioxidant properties. The abundant phytochemicals are flavonoids, phenolic glycosides, coumarins, and, especially, isoflavones, which makes *T. pratense* compete with soy for the most affordable botanical rich in phytoestrogens [19]. While soy contains mostly glycosidic isoflavones (e.g., genistin, daidzin), the red clover includes aglycones forms, mainly biochanin A and formononetin, which are the methylated derivates of genistein and daidzein. Moreover, aglycones are absorbed and metabolized approximately three times better than glycosidic compounds [20]. Thus, red clover-based food or supplements can have a greater impact on humans than soy. 

Currently, the *T. pratense* supplements are recommended as alternative hormone replacement therapy, claiming to alleviate pre- and postmenopausal syndromes. However, the clinical evidence of its efficacy is still in dispute. The standardized extracts of red clover isoflavones, at a daily dose of 80 mg, have demonstrated the capacity to reduce hot flushes in menopause [21] and can positively influence the lipid profile in peri- and postmenopausal women [22]. On the other hand, other studies have suggested a very small positive health effect with uncertain clinical relevance in different human pathologies, such as cardiovascular and osteoporotic markers and nervous system disorders [23]. Moreover, due to red clover’s ability to interfere with endogenous estrogen activities, there are concerns about red clover supplements which may produce adverse effects in the case of premalignant hormone-sensitive conditions, particularly in the breast cancer risk population.

Several in vitro studies have revealed that the extracts prepared from different parts of red clover were capable of inducing cytotoxicity and inhibiting tumorigenic cells proliferation, such as breast cancer, hepatocarcinoma, and glioblastoma. The cytotoxic effect was found to be correlated with induced apoptosis and autophagy in glioblastoma and breast cancer cells, leading to reduced cell viability in a time- and dose-dependent manner [24,25,26]. Additionally, red clover extracts have been shown to inhibit angiogenesis and inflammation, which are key processes involved in cancer progression [27]. The antioxidant and free radical scavenging properties of red clover compounds may also contribute to plant extract chemoprevention potential by protecting cells from oxidative stress damage that can lead to cancer. Furthermore, phytoestrogens present in *T. pratense* have been proven to act as antioxidants and possess tyrosine kinase inhibitory activity [28]. As potent aryl hydrocarbon receptor (AhR) agonists, biochanin A and formononetin can transactivate the receptor and influence the ER signaling, and thus, in certain contexts, they might sustain tumor growth [29].

There are few in vivo studies on red clover extracts, most of them examining the estrogenic effect induced by administration on ovariectomized rat models. A red clover extract standardized with 15% isoflavones produced a dose-dependent growth in uterine weight and increased vaginal cell differentiation, yet it did not stimulate mammary gland cell proliferation [30]. The anti-inflammatory potential of a 40% ethanolic extract of *T. pratense* leaves was demonstrated on LPS-treated murine macrophages and in vivo on a carrageenan-induced inflammation model. Pretreatment with extract significantly inhibited inflammatory cytokines, like tumor necrosis factor (TNF)-α and interleukin (IL)-1β, by suppressing the nuclear translocation of nuclear factor (NF)-κB and the phosphorylation of mitogen-activated protein kinases (MAPKs). Oral administration of extract reduced carrageenan-induced edema in a concentration-dependent manner by inhibition of inflammatory mediators such as histamine and serotonin [27].

Overall, the available evidence suggests that *T. pratense* extracts exhibit promising anticancer and chemoprevention effects, particularly through their ability to inhibit cancer cell proliferation, as well as reduce inflammation, oxidative stress, and angiogenesis. However, there are no reports exploring possible biphasic dose effects of *T. pratense* extracts on breast cell proliferation and oxidative stress, knowing that it is a plant rich in phytoestrogens and compounds with antioxidant properties. In this study, we aimed to perform the phytochemical characterization of two red clover extracts and investigate their biological effects on cell proliferation and viability and examine the extracts’ capacity as antioxidants or as generators of ROS on human breast normal and malignant cells. To the best of our knowledge, this is the first study investigating the possible pro-oxidant effect of *T. pratense* extracts on breast normal ER-insensitive cells, adenocarcinoma ER-positive cells, and highly invasive adenocarcinoma ER-negative cells.

## 2. Materials and Methods

### 2.1. Chemicals and Reagents

All the reagents and chemicals were of analytical grade and were purchased from the following companies: Sigma-Aldrich (St. Louis, MO, USA) provided gallic acid 100%, anhydrous sodium carbonate, 2,2-Diphenyl-1-picrylhydrazyl ACS reagent; VWR Chemicals (Radnor, PA, USA): Folin—Ciocalteu reagent, sodium acetate trihydrate, sodium nitrite, sodium hydroxide, ferrous sulfate heptahydrate; Merck (Darmstadt, Germany): butylated hydroxytoluene (purity ≥ 99%); Acros Organis (Geel, Belgium): neocuproine (2,9-dimethyl-1,10-phenanthroline), TROLOX ((±)-6-Hydroxy-2,5,7,8-tetramethylchromane-2-carboxylic acid); Alfa Aesar (Haverhill, MA, USA): anhydrous aluminum chloride, ammonium acetate, anhydrous iron (III) chloride; Cayman Chemical (Ann Arbor, MI, USA): quercetin standard; PanReac AppliChem (Darmstadt, Germany): Cu (II) sulfate pentahydrate, methanol ≥ 99.8%, acetonitrile ≥ 99.9%, formic acid ≥ 98%, all of them with HiPerSolv CHROMANORM^®^ or Ph. Eur, standards. The isoflavones standards: daidzein (≥98%), genistein (≥98%), formononetin (≥98%), and biochanin A (≥98%), were purchased from Shanghai Juyuan Biological Technology Co., Ltd. (Shanghai, China).

### 2.2. Plant Material and Extracts Preparation

The plants were collected from the cultivated lands of Hofigal SA in September 2021. The aerial parts were harvested in order to obtain adequate amounts for physicochemical analysis and in vitro studies. Both leaves and flowers were examined to verify the absence of contamination and their integrity. The aerial parts were dried for 48 h at a temperature of 35 °C (Dehydrator Drying Oven, Biovita, DELUXE-10) and, then, were powdered using a mill grinder (GRINDOMIX GM 200, Verder Scientific, Secunderabad, India).

The aqueous extract of *Trifolium pratense* (Tp-aq) was prepared by heat reflux extraction (HE) performed for 30 min at a temperature of 80 °C using 5 g of powder raw material and 50 mL solvent (ultra-purified water). After reflux, the sample was left to cool down until it reached a temperature of 25 ± 2 °C. Next, the sample was centrifuged for 10 min at 7800× *g*, followed by the decantation of the supernatant.

The ethanolic extract of *Trifolium pratense* (Tp-eth) was obtained by ultrasound-assisted extraction (UAE) using an ultrasound bath (Elmasonic, P70H, Elma Schmidbauer GmbH, Singen, Germany). The same ratio of plant material to solvent 1:10 (*w*/*v*) was used, and the solvent was a solution of 50% ethanol prepared in distilled water. The extraction time was 30 min with a processing frequency of 50 kHz at a temperature of 60 ± 2 °C. After the extraction, the mixture was left to cool down and then centrifuged for 10 min at 7800× *g*, followed by decantation of the supernatant. After the filtration through PVDF syringe filters (pore size 0.22 μm), both extracts were kept at +4 °C until use. Fresh extracts were prepared from the same batch of dried plants whenever needed.

### 2.3. Determination of Total Phenolic Content (TPC)

The total phenolic content of *T. pratense* extracts was quantified using the method described by [31] with slight modifications. Briefly, a volume of 1 mL of extract solution was mixed with 5.0 mL of 1:10 diluted Folin–Ciocalteu’s phenol reagent and 4 mL of 7.5% (*w*/*v*) sodium carbonate. Absorbance was measured at a wavelength of 765 nm (Jasco V-530 UV-Vis Spectrophotometer, Jasco Corporation, Tokyo, Japan) after 1 h of incubation of the reaction mix. The total phenolic content was expressed as mg gallic acid equivalents per gram extract (mg GAE/g extract).

### 2.4. Determination of Total Flavonoid Content (TFC)

The total amount of flavonoids from plant extracts was measured as quercetin equivalents using the aluminum chloride colorimetric method [32]. In summary, the sample extracts were mixed with 4 mL distilled water and 0.3 mL of 5% sodium nitrite solution, then incubated for 5 min at room temperature. A volume of 0.3 mL of 10% aluminum chloride was added and the mixture was incubated for another 6 min at room temperature. Finally, 2 mL of 1 M sodium hydroxide was added, and the volumes were brought up to 10 mL with distilled water. The absorbance was measured at 510 nm using a UV/Vis spectrophotometer (Jasco V-530 UV-Vis Spectrophotometer, Jasco Corporation, Tokyo, Japan).

### 2.5. HPLC-UV Method for Isoflavones Analysis

The analysis of isoflavones from *T. pratense* extracts was carried out using an in-house method developed after the validated HPLC method described by Kumar et al. [33] and was performed on VWR Hitachi Chromaster HPLC System (Hitachi, Tokyo, Japan) equipped with a Chromolith^®^ HighResolution RP-18 endcapped 100-4.6 HPLC column (Merck, Darmstadt, Germany). In summary, samples of 1.5 g of each extract were mixed with methanol at a 1:10 (*w*/*v*) ratio and then ultrasonicated for 10 min at 80 kHz. An aliquot of 5 μL was injected into the HPLC for analysis. The gradient system was used for isoflavone separation, with methanol, acetic acid 0.1%, and acetonitrile as mobile phases. The flow rate was also gradient 1.5–3.5 mL/min. The effluents were monitored at 255 nm with a DAD 5430 detector at ambient temperature. All four isoflavones (daidzein, genistein, formononetin, and biochanin A) from samples were identified by comparing their retention times and UV spectra with those of the corresponding standards. The standard solutions were prepared in methanol and a good linearity (R^2^ > 0.999) was obtained with 5 concentrations of each isoflavone within the range of 19.8–99 μg/mL (daidzein), 9.6–48 μg/mL (genistein), 7.4–373.4 μg/mL (biochanin A), and 39.6–198 μg/mL for formononetin.

### 2.6. Antioxidant Activity Evaluation

#### 2.6.1. DPPH Scavenging Activity Assay

Briefly, a solution of 0.5 mM DPPH in methanol and 0.1 M acetate buffer was mixed with the sample dissolved in methanol. After 30 min incubation at room temperature, the absorbance of the mixture at 517 nm was measured using a JASCO V-530 UV/VIS Spectrophotometer (Jasco Corporation, Tokyo, Japan) [34]. Butylated hydroxytoluene (BHT) was used for the calibration curve, and the results are presented as mg BHT equivalent/gram extract.

#### 2.6.2. Ferric Reducing Antioxidant Power (FRAP) Assay

The experiment followed the technique described by Csepregi et al. [35], with several alterations. A freshly prepared FRAP reagent was mixed with samples dissolved in methanol (100–500 μg/mL) to reach a volume of 2 mL, which consequently was refilled with distilled water to a final volume of 10 mL. The mixture was incubated at 37 °C temperature for 10 min, then the absorbances were measured at 593 nm using a JASCO V-530 UV/VIS Spectrophotometer (Jasco Corporation, Tokyo, Japan). The results were expressed as mg Fe^+2^ equivalent/gram extract.

#### 2.6.3. Cupric Ion Reducing Antioxidant Capacity Assay (CUPRAC)

The antioxidant activity was measured using the CUPRAC assay, as described by Munteanu et al. [34]. The antioxidant activity was calculated using a Trolox standard calibration curve and expressed as mg Trolox equivalent per gram extract.

### 2.7. Cell Culture

All cell lines were purchased from the American Type Culture Collection (ATCC): MCF-12A (CRL-3598™) human breast epithelial non-tumorigenic cell line; MCF-7 (HTB-22™) human breast adenocarcinoma cell line, and MDA-MB-468 (HTB-132™) human breast metastatic adenocarcinoma cell line. MCF-12A cells were cultured in Dulbecco’s modified Eagle medium/nutrient mixture F-12 (DMEM:F12) supplemented with 20 ng/mL of epidermal growth factor, 500 ng/mL of hydrocortisone, 0.01 ng/mL of insulin, 5% horse serum, and 1% penicillin/streptomycin mixture, all from Gibco (Waltham, MA, USA). The MCF-7 cells were cultured in RPMI-1640 medium (Sigma-Aldrich, St. Louis, MO, USA) and MDA-MB-468 cells in high glucose DMEM media (Gibco, Waltham, MA, USA); the culture media were supplemented with 10% bovine serum (Sigma-Aldrich, St. Louis, MO, USA) and 1% penicillin/streptomycin solution (Sigma-Aldrich, St. Louis, MO, USA). For E-screen experiments, the cell media without phenol red was used: RPMI-1640 (Lonza Bioscience, Walkersville, MD, USA) for MCF-7 cells and DMEM:F12 (Thermo Fisher Scientific, Waltham, MA, USA) for MCF-12A cells. The normal serum was replaced by charcoal-stripped fetal bovine serum from Sigma-Aldrich (St. Louis, MO, USA). The cells were maintained in standard conditions such as a humidified atmosphere with 5% CO_2_ and 37 °C.

### 2.8. Cytotoxicity of Plant Extracts Was Assessed by Cell Viability Test (MTS) and by LDH Released Test

The cell viability test was performed according to the Technical Bulletin of Promega Corporation (Madison, WI, USA), CellTiter 96 Aqueous One Solution Cell Proliferation Assay, as described previously [36]. Briefly, cells were placed for 24 h on 96-well plates at a density of 10,000 cells/well (MDA-MB-468) and at a density of 8000 cells/well (MCF-12A and MCF-7). Next, the culture media were changed and new media with the plant extracts at the corresponding concentration were added for 48 and 72 h of treatment. The assay was performed by adding 20 μL/well of MTS reagent directly to culture wells, incubating for 1–4 h, and then recording the absorbance at 490 nm with a BIOBASE-EL10 microplate reader (Jinan, China). The cell viability was presented as a percentage by reporting the normalized OD 490 nm reading of samples to the normalized OD 490 of control, untreated cells. The results are expressed as a percentage of cell viability of the treated sample compared with untreated cells (negative control—considered as 100% of cell viability for each type of the cell line).

The LDH test for measuring the cytotoxicity of plant extracts was assessed using CytoTox 96^®^ Non-Radioactive Cytotoxicity Assay (Promega Corporation, Madison, WI, USA). The lactate dehydrogenase (LDH) amount released in the culture medium measures the cell membrane integrity. According to the manufacturer’s instructions, a lysis solution was used to generate a maximum LDH release, which was considered the positive control and was determined for each type of cell used in this study. The reaction was stopped by adding 50 µL of a stop solution, and the absorbance was read at 490 nm using a BIOBASE-EL10 microplate reader (Jinan, China). The cytotoxicity is presented as a percentage by reporting the LDH release from the sample to the total amount of LDH present in lysed cells.

Each treatment (cell series treated with plant extracts at different concentrations) was carried out in triplicate and the experiments were repeated at least three times. The experiments with cells treated with vehicle alone (ultrapure water and 50% ethanol–water solution), at the corresponding concentrations, were performed, and no significant variation in comparison with untreated cells (control) was observed for both tests.

### 2.9. Real-Time Cellular Proliferation Monitoring

The proliferation of the cells was monitored in real time using the xCELLigence RTCA system (Agilent, San Diego, CA, USA). This system enables continuous monitoring of the adhesion properties of cells by detecting variations in electrical impedance that are transformed into a dimensionless factor referred to as the cell index (CI) [37]. The CI was recorded every 30 min for approximately 7 days. Briefly, a number of 6000 cells (MCF-7 and MDA-MB-468) and 4000 cells (MCF-12A) were plated in a 96-well format on the E-Plate and their growth rate was monitored 24 h prior to treatment. Then the medium was changed and new culture media with the corresponding treatment were added (total volume of 200 µL/well). Control samples were cells with media without treatment. All the samples and controls were tested in triplicates for each experiment. Three independent experiments were performed. For each experiment, an additional control of media with extract solvent at the highest concentration was added. No significant differences were observed for cells treated with vehicles at concentrations used in the present study. The CI was normalized to the time point of plant extract addition. Growth curves for each treatment and control were obtained and compared using RTCA software (Version 2.0).

### 2.10. E-SCREEN Assay

The estrogenic activity of plant extracts was evaluated using an E-screen assay [38] with some modifications. The MCF-7 and MCF-12A cells exposed to the corresponding steroid-free experimental medium served as negative control, while cells treated with 1 nM of 17β-estradiol (E2) from Sigma-Aldrich (St. Louis, MO, USA) were positive control. A number of 6000 cells for MCF-7 and 4000 cells for MCF-12A were seeded in each well of a 96-well plate. The cells were treated with the indicated concentrations of the plant extracts for 6 days. For samples co-treated with plant extracts and ER antagonist, fulvestrant, ICI 182,780 (Abcam, Cambridge, UK) was added to reach a final concentration of 100 nM. To estimate the cell viability (proliferation) after 6 days of treatment, the MTS kit was used as described above.

### 2.11. Cellular Antioxidant Activity Evaluation

The OxiSelect™ Cellular Antioxidant Assay Kit (Cell Biolabs, San Diego, CA, USA) was used to assess the cellular antioxidant activity of plant extracts, following the manufacturer’s instructions, with some modifications. The MCF-12A and MCF-7 cells were plated into 96-well plates at a cell density of 40,000 cells/well. Twenty-four hours later, the cells were washed with PBS and incubated overnight with plant extracts at different concentrations. The next day the cells were washed in PBS 1x and incubated for 1 h with DCFH-DA solution. A 100 µL volume of the free-radical generator, namely, 2,2′-azobis (2-amidinopropane) dihydrochloride (AAPH) solution, was added to testing wells in order to generate intracellular free radicals. The background signal for each plant extract treatment was considered the fluorescence recorded on the cells untreated with a free radical generator; instead, PBS 1× solution was added. All samples were analyzed in triplicate and the experiments were repeated three times. The fluorescence was recorded for 1 h, at every 10 min with Microplate Multimode Detector Zenyth 3100 (Anthos Labtec Instruments GmbH, Salzburg, Austria). The results were calculated according to the instructions presented by the manufacturer and were represented as ROS production (%) reported to control cells without plant extract treatment, which generated the maximum ROS in the presence of AAPH solution.

### 2.12. Cellular NO Scavenging Capacity Evaluation

The cells were stimulated to produce NO radicals by treatment with sodium nitroprusside dihydrate (Sigma-Aldrich, St. Louis, MO, USA) as described [39]. The sodium nitroprusside dihydrate (SNP) stock solution was prepared in distilled water at a 50 mM concentration. A number of 2 × 10^5^ cells per well on a 24-well plate were placed the day before the experiment. The cells were pretreated overnight with plant extracts at the noted concentrations. Cells were washed with PBS 1× and then exposed for 6 h to SNP solution (1 mM) in culture media without phenol red. The NO scavenging activity of plant extracts was evaluated in each type of cell by measuring the quantity of nitrite released into the supernatant culture media using the Griess reagent system (Promega Corporation) and following the manufacturer’s instructions. The spectrophotometric readings at 540 nm were performed on a BIOBASE-EL10 microplate reader (Jinan, China). The positive control sample was represented by cells incubated just with SNP solution to assess the maximum quantity of nitrite released. The experiments were conducted three separate times, and each time the samples were worked in quadruplicates. The amount of nitrite was calculated from a NaNO_2_ standard curve (0–200 μM). The results were presented as a percentage of nitrite production (%) reported to the positive control sample.

### 2.13. Intracellular ROS Detection by Time-Lapse Imaging

Reactive oxygen species (ROS) were detected using the CellROX Green reagent (Life Technologies, Molecular Probes, Carlsbad, CA, USA). This reagent is a cell-permeant dye that is weakly fluorescent while in a reduced state. Once inside the cell, the dye is oxidized by ROS and becomes highly fluorescent with an absorption/emission maxima at ~485/520 nm. The level of fluorescence is proportional to the amount of ROS present in the cells and the signal can be measured using live imaging techniques. Approximately 40,000 cells were plated on each compartment of µ-dish 35 mm quad (Ibidi, Gräfelfing, Germany). The cells were kept for 24 h to attach, then washed with PBS 1×, and new media supplemented with 5 μM CellROX Green reagent were added and left to penetrate the cells for 45 min. Finally, the cells were placed in new media supplemented with plant extracts at different concentrations. The vehicle at the highest concentration corresponding to 3.33 mg/mL was tested and no differences in ROS generation in comparison with untreated cells were observed. In all experiments, one out of four compartments of Ibidi µ-dish was kept with cells without treatment as a control sample. The Ibidi µ-dishes were placed into BioStation IM (Nikon, Tokyo, Japan), which is a platform for live cell imaging. Phase contrast and green channel images were taken every 1 h for approximately 24 h of cells under the treatment with plant extracts. Therefore, the oxidative stress induced by plant extracts was detected by time-lapse imaging and quantified in live cells in real time. Each experiment was repeated three times and the images were analyzed by the ImageJ program (version 1.50i, National Institutes of Health, Bethesda, MD, USA).

### 2.14. Determination of ATP Levels

The ATP levels were measured using an ATP determination kit (Invitrogen™, Thermo Fisher Scientific, Waltham, MA, USA). Cells were seeded into 6-well plates at a density of 200,000 cells/well in 1 mL volume of corresponding cell culture media and allowed to grow overnight. The next day, media were replaced with media supplemented with plant extracts at desired concentrations, and the cells were incubated for 48 h. Following the manufacturer’s instructions with slight modifications, the cells were lysate using an ATP assay buffer supplemented with 1% Triton X-100. The cells were dislodged by scraping with a pipette tip and transferred to 1.5 mL Eppendorf tubes on ice for 5 min. A volume of 10 μL of cell lysate was then added in triplicate to a white 96-well plate, containing ATP standards. The luminescence was recorded with Microplate Multimode Detector Zenyth 3100 (Anthos Labtec Instruments GmbH, Salzburg, Austria). Protein concentration was determined using the Pierce 660 nm Protein Assay (660 nm absorbance). The results were presented as ATP level/mg protein and reported to the ATP level of the control sample. The experiments were repeated three times.

### 2.15. Mitotracker and Cellrox Staining

Cells seeded at 40,000 cells/well of µ-Dish 35 mm Quad (Ibidi, Gräfelfing, Germany) were treated for 48 h with plant extracts. Then, the cells were washed with PBS 1× and were double-stained with a mixture of CellROX green reagent (5 μM) for ROS staining and 150 nM of MitoTracker^®^ red CMXRos (Thermo Fisher Scientific, Invitrogen, Waltham, MA, USA) solution for mitochondria visualization. -The protocol followed the manufacturers’ instructions, and after the 30 min incubation at 37 °C, the cells were fixed in 4% formaldehyde for 30 min. The cells were washed in PBS and were immediately observed under the microscope (Eclipse Ti, Nikon, Tokyo, Japan). Approximately 200 cells were observed for each experimental condition in one experiment setting. The experiments were repeated three times. The MitoTracker Red^®^ CMXRos reagent can accumulate in active mitochondria and is retained even after formaldehyde fixation, thus being well suited for multicolor labeling experiments. The CellROX green and MitoTracker^®^ red CMXRos signals were analyzed by using Image J software (version 1.50i, National Institutes of Health, Bethesda, MD, USA).

### 2.16. Assessment of Lipid Peroxidation

Lipid peroxidation assessment was measured as MDA content in cells using the OxiSelect™ TBARS Assay Kit (MDA Quantitation kit), from Cell Biolabs (San Diego, CA, USA). The protocol provided by the manufacturer was followed with slight modifications. A number of 1 × 10^7^ cells were used for each experiment, and after 48 h of incubation with plant extracts, the cell homogenates were resuspended in 1 M HCl solution. Thus, in the necessary acidic environment (pH 4) the MDA and thiobarbituric acid can react and form the final product that was quantified fluorometrically at 540 nm excitation and 590 nm emission by Microplate Multimode Detector Zenyth 3100 (Anthos Labtec Instruments GmbH, Salzburg, Austria). The protein concentration was determined using Pierce 660 nm Protein Assay. Data were calculated as µmol MDA/g protein and reported as percentages to control, untreated cells. The experiments were repeated three times.

### 2.17. Assessment of SOD Activity and GSH Content

A number of 2 × 10^6^ cells were used for each experimental condition, for both types of assessment. The cells were incubated for 48 h with Tp extracts at 0.33 and 3.33 mg/mL, and then, the specific determination for each assay was performed.

The activity of SOD was detected by the commercial SOD Determination Kit from Sigma-Aldrich (St. Louis, MO, USA). The kit’s protocol was followed and the cells’ supernatant was used for total SOD (cytosol and mitochondria) activity assessment. The absorbance of samples was read at 450 nm using a BIOBASE- EL10 microplate reader (Jinan, China). The protein concentrations were determined by Pierce 660 nm Protein Assay (Thermo Fisher Scientific, Waltham, MA, USA) using BSA as standard. The results were expressed as a percentage of SOD activity reported to protein concentration (mg/mL) and then compared with control (untreated cells) values.

The OxiSelect™ Glutathione Reductase Assay Kit (Cell Biolabs, San Diego, CA, USA) was used for measuring glutathione reductase activity. The kit employs the enzymatic recycling reaction for glutathione quantification, and the reduction in the chromogen compound is correlated to glutathione reductase enzymatic activity. The manufacturer’s instructions were followed, and the optical density of samples and glutathione reductase standards were recorded at 405 nm (BIOBASE- EL10 microplate reader, Jinan, China). The protein concentration of samples was analyzed as above and the results were expressed as a percentage of GSH level reported to protein concentration (mg/mL) and then to the GSH level of control (untreated cells), which is considered 100%. All samples were analyzed in triplicate for one experiment setting, and the experiments were repeated three times.

### 2.18. Morphological Assessment of Cell Death Using Hoechst 33342/Propidium Iodide (PI) Staining and Fluorescence Microscopy

A number of 1 × 10^6^ cells/well were seeded for plant extract treatment and for control (untreated cells) in 6-well plates. After 48 h of treatment, all cells were collected and double-stained with 1 μM Hoechst 33342 dye (Thermo Fisher Scientific, Waltham, MA, USA) and with 50 µg/mL propidium iodide (PI) from Sigma-Aldrich (St. Louis, MO, USA), as previously described [40]. The cells were immediately observed under the fluorescent microscope (Nikon Eclipse Ti, Tokyo, Japan), and random images of different fields were collected in blue and red channels and phase contrast. A minimum of 200 cells for each sample/experiment were analyzed using the Image J cell counter software (version 1.50i, National Institutes of Health, Bethesda, MD, USA). The experiments were repeated three times.

### 2.19. Statistical Analysis

Data are presented as means ± SEM. One-way ANOVA analysis was used to compare all groups with control conditions (as indicated in the Figure legends) using a Dunnett post-test. Statistical significance is indicated as * *p* < 0.05, ** *p* < 0.01, *** *p* < 0.001.

## 3. Results

### 3.1. Total Phenolic and Flavonoids Content of T. pratense Extracts

The values obtained for TPC and TFC assessments are presented in Table 1. The Tp-eth extract contains the highest amounts of polyphenols extract in comparison with Tp-aq. Our results are aligned with the findings of other studies, which reported values of TPC in a range of 5.22–8.38 mg GAE/g extract for Romanian *T. pratense* obtained by UAE using different parameters and as solvent a 70% ethanol solution [41]. Less polyphenolic content as 46.56 mg GAE/100 g d.w. was obtained by Antonescu et al., using ultrasonication for 5 min and 70% ethanol solution as solvent [42]. The flowers and leaves of six different varieties of *T. pratense* from Poland were analyzed and the results showed significant differences of TPC values ranging from 12.758 to 56.178 mg GAE/g d.w., with more polyphenolic content in flowers compared with leaves [43]. In general, the pre-extraction drying condition of original plant material could influence the total polyphenolic content. The polyphenol oxidases, the enzymes which are degrading the polyphenolic compounds, can be inactivated by drying at temperature above 55 °C [44]. In the present paper, the aerial parts (flowers, leaves, stems) were dried at 35 °C for 48 h, so the polyphenol oxidases were still active and could degrade the polyphenols, resulting in a decrease in TPC. Specifically for *T. pratense*, Schmitz et al. demonstrated that polyphenol oxidases can be activated with a 10- to 40-fold increase in enzymatic activity by long incubation of the plant at ambient temperature [44].

The total flavonoids analysis demonstrated that the ethanolic extract contains approximately twice as much flavonoids as Tp-aq extracted by heat reflux method, as Table 1 shows. The values obtained in the present study were approximately similar to those obtained in other studies using Romanian red clover aerial parts extracted by UAE in 70% hydro-alcoholic solvent [41,42]. Moreover, Vlaisavljević et al. [45] showed that the TFC values of leaves from plants at different growing stages varied between 3.87 and 7.32 mg QE/g of extract. They applied the microwave-assisted extraction and a 70% methanol solution was used as a solvent, demonstrating that the highest flavonoid content was present at the lowest red clover growth stage [45]. Others reported much higher values for TFC, such as 18.17 ± 0.67 or 20.51 ± 0.39 mg RU/g d.w., in flowers extracted by heat reflux method, using water or 50% ethanol as solvents [46], or even lower values close to 15.0 μg rutin equivalents/g extract of the aqueous phase obtained from aerial parts prepared by maceration in 80% methanol [28].

The higher values of TPC and TFC obtained for Tp-eth were determined by the extraction method. UAE is a nonconventional method, which allows for a rapid extraction due to an increase in the permeability of cell walls. Furthermore, the binary solvent, 50% ethanol in water, is more successful in the extraction of polyphenols, including flavonoids, than water as a single solvent [47]. UAE is recognized as a more effective technique to increase the yields of polyphenols than other traditional methods, including heat reflux extraction [47]. Beyond quantity, the profile of present species will also vary according to the extraction method used (regardless of the analyzed matrix).

### 3.2. HPLC Analysis for Isoflavones Content of T. pratense Extracts

**Figure 1:**
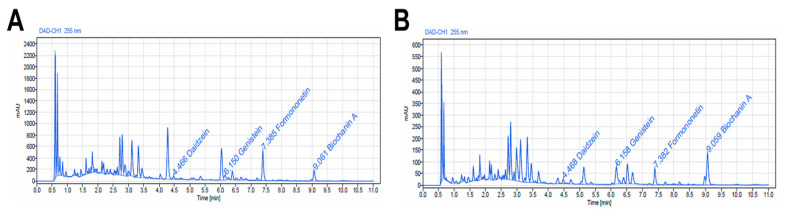
The representative HPLC chromatograms obtained for Tp-aq (**A**) and Tp-eth (**B**) extracts.

The representative HPLC chromatograms of both extracts, showing the separated isoflavones in aglycone form, are presented in Figure 1.

The main isoflavonoid compounds found in *T. pratense* extracts were formononetin, biochanin A, daidzein, and genistein. The Tp-eth extract obtained by UAE method contains a slightly higher amount of total isoflavones, such as 636.43 ± 16.2 μg/g extract in comparison with Tp-aq, which had 603.94 ± 13.7 μg/g, as Table 2 shows.

While daidzein was recovered approximately in the same amount in both extracts, the genistein concentration was 2.5 times higher in Tp-eth compared with Tp-aq. In the case of daidzein, our results were similar to those obtained by Gligor et al., using aerial parts of Romanian *T. pratense* and different types of extraction, and by analyzing the isoflavones with a more sensitive method, HPLC-MS [41]. They reported that UAE applied for 20 min at a temperature of 50 °C and with a frequency of 50 Hz led to the highest yield of isoflavones such as daidzein, genistein, and their glycosidic correspondents [41]. Approximately the same amount of genistein was reported by Kazlauskaite et al., who also demonstrated that the two isoflavones, genistein and daidzein, can be extracted in aglycone forms without any extra step of chemical hydrolysis [48]. Indeed, the parameters for extractions used in the present paper allow for the spontaneous conversion of glycosides to aglycones by β-glucosidase, which is active in red clover grounded powder at high temperatures and during the ultrasonication treatment. Overall, our results show that the amount of daidzein is constant in both extracts, but the genistein is more abundant than daidzein and it is present in higher concentration in Tp-eth in comparison with Tp-aq. This can be explained by the fact that genistein is one of the isoflavones that are more susceptible to degradation at high temperatures, including 80 °C, which was the temperature for Tp-aq preparation [49]. In contradiction, formononetin, which is a derivative of daidzein with a methoxy radical in the 4′ position seemed to be more reliably extracted by HE using water as a solvent than by UAE in 50% water–ethanol mixture (369.46 vs. 139.76 μg/g). The concentration was approximately twice as high in the ethanolic extract as in the aqueous one. Our data are comparable with those reported by Temerdashev et al. [50] where the maximum amount of isoflavones extracted from flowers using the supercritical method was 598 μg/g extract [50], which is very close to the value obtained in the present study. Moreover, the approximately similar ratio of daidzein, genistein, and formononetin was recovered from Romanian *T. pratense* of spontaneous flora extracted by UAE, with the mention that biochanin A was not included in the HPLC-UV analysis [51]. There are reports showing the same pattern of the presence of four isoflavones in extracts obtained from flowers or leaves of red clovers collected from all over of Europe [50,52]. Biochanin A and formononetin made up over 70% of total isoflavones [52], very similar to what was obtained in the present paper.

### 3.3. Antioxidant Capacity of T. pratense Extracts Evaluated by Chemical-Based Assays

The antioxidant capacity of Tp extracts was established using three different chemical-based assays. It is highly recommended to assess the antioxidant potential of plant extracts by different methods, given their complex composition consisting of many different classes and types of chemical compounds [34,53]. The selected methods concern different ways of testing antioxidant potential, and herein, a free radical scavenging assay (DPPH), ferric ion reducing antioxidant power (FRAP), and cupric-reducing antioxidant capacity (CUPRAC) were used. The DPPH assay measures the capacity of test samples to reduce DPPH radical to DPPH-H by transferring atoms of hydrogen and, consequently, decreasing the absorbance of the reaction mixture [53]. This method has some drawbacks, and one of them is that the compounds with strong antioxidant capacities in lipid peroxidation may react slowly or even will not react with DPPH radicals [34]. The second test, FRAP assesses the antioxidant compounds as reductants in a redox-linked colorimetric method where at low pH (3.6), the Fe^3+^ is reduced to Fe^2+^ by electron transfer [53]. In this method, the increase in absorbance is directly related to the reducing power of the electron-donating antioxidants present in the reaction solution. In general, the FRAP assay detects hydrophilic antioxidants (e.g., hydroxybenzoic and hydroxycinnamic acids) that complete the reaction rapidly, within 10 min. However, it has been demonstrated that several other polyphenols are slow-reacting substances, (e.g., caffeic, gallic, or tannic acid) that can be totally oxidized after several hours. Thus, in the case of a higher amount of those slowly reacting compounds, the antioxidant capacity of plant extracts will be under-evaluated by the FRAP assay. The cupric-reducing antioxidant capacity assay is similar to the FRAP method. CUPRAC measures the capacity of plant extracts to reduce Cu (II) to Cu (I) as a result of a redox reaction by electron transfer. This method could be applied to the quantification of both hydrophilic and lipophilic antioxidants because the Cu (II) reagent is soluble in both aqueous and organic environments [34]. Additionally, the CUPRAC assay works at physiological pH = 7, as opposed to FRAP and Folin tests, which are completed at pH 3.6 and pH 10, respectively. Moreover, the assay can measure the reducing power of glutathione and nonprotein thiols, so the thiol-type antioxidants are also quantified [34]. For some specific compounds, including caffeic, chlorogenic, gallic, and rosmarinic acids, catechin, epicatechin, quercetin, and rutin, the best antioxidant capacities were observed using this method [34].

The results of antioxidant capacity evaluation based on all three methods are presented in Table 3.

The highest values of antioxidant capacity were obtained for the ethanolic extract in comparison with the aqueous extract by all three methods, as was expected. The CUPRAC assay showed notably higher values than the other two assays, indicating that the red clover extracts contain many compounds with optimal number and orientation of hydroxyl groups for transferring the electrons to reduce Cu (II) reagent [34]. The results reported by Antonescu et. al. for a 70% hydro-alcoholic extract were similar to what was obtained in this study for Tp-eth [42]. Other reports showed lower values with the CUPRAC method, with values ranging from 0.644 to 3.363 mg/mL, with the mention that the antioxidant capacity of leaves and flowers of different species of *T. pratense* were analyzed and the standard substance was ascorbic acid instead of Trolox as was used in the present paper [43].

A previous study reported values between 9 and 10 μg Trolox equivalents/g for red clover aqueous extracts and between 10 and12 μg Trolox equivalents/g for ethanolic extracts in DPPH assay for flowers of Lithuanian *T. pratense* [46]. Moreover, compared with the DPPH values obtained from other studies, our results were significantly higher, indicating that water or ethanol as solvents and the methods of extraction were suitable for recovering the compounds with strong scavenging capacity from red clover aerial parts [25,46]. In contrast, the FRAP assay results showed that both extracts had the weakest ability to reduce Fe^3+^, with values of 1.96 ± 0.08 for Tp-aq and 2.26 ± 0.11 mg Fe^2+^/g for Tp-eth, notably lower than results reported by others [46,54]. However, there are scientific papers that presented a much lower reducing antioxidant power, as 0.0251 mg Fe^2+^/g d.w. [55] or even undetected FRAP activity [56] for red clover flower extracts. This means that the presence of phenolic compounds with different antioxidant capacities and their distribution in the *T. pratense* could vary due to many factors—the origin of species, cultivar conditions, growing stages, harvesting season, and so on, as mentioned before [57,58].

### 3.4. Cytotoxic Activity of T. pratense Extracts and the Effect on Cells Viability

**Figure 2:**
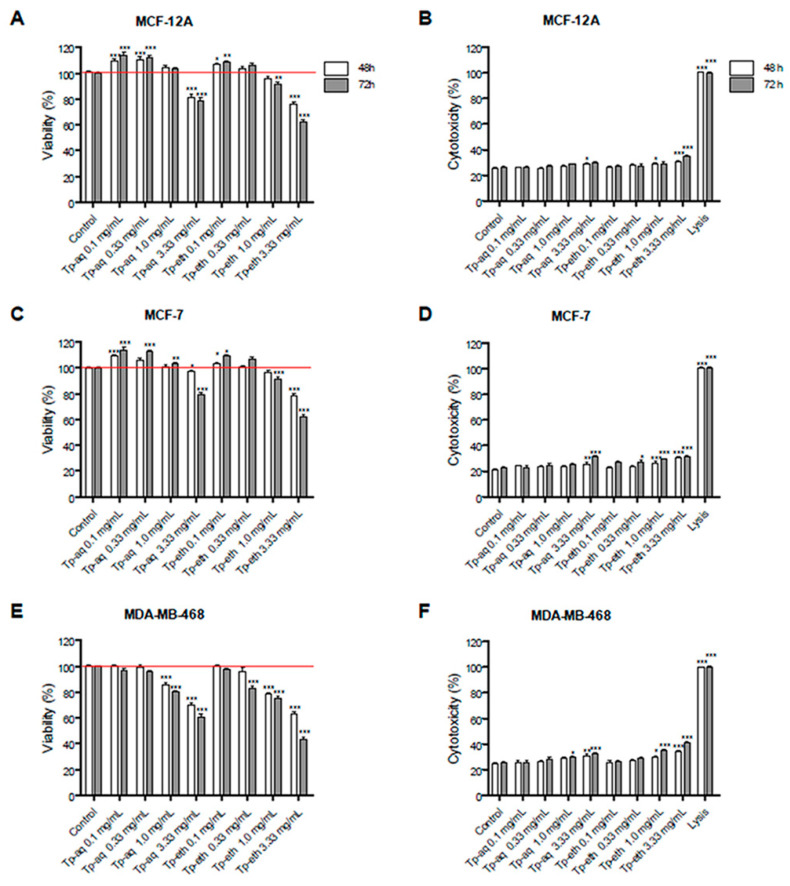
Cell viability (%) and cytotoxicity (%) expressed based on the MTS test and, respectively, as LDH released on MCF12A cells (**A**,**B**), MCF7 cells (**C**,**D**), and MDA-MB-468 cells (**E**,**F**) treated with Tp-aq or Tp-eth for 48 and 72 h. The red line indicates 100% cell viability. The results represent the mean ± SME of at least three independent experiments with *p* < 0.05 (*), *p* < 0.01 (**), and *p* < 0.001 (***) compared with control (untreated cells for cell viability and to complete lysed cells for cytotoxicity).

The cytotoxic activity of both extracts was evaluated on breast non-tumorigenic ER- cell line (MCF-12A), breast estrogen-dependent (ER+) cells (MCF-7), and metastatic estrogen-independent (ER−) adenocarcinoma cells using MTS and LDH assays. All three cell lines were tested for 48 and 72 h of treatment with aqueous and ethanolic extracts of *T. pratense* at concentrations ranging between 0.1 to 3.33 mg/mL. The cell viability of all three cell lines was affected by Tp-eth extract at the highest concentration of 3.33 mg/mL in a time-dependent manner (Figure 2). The adenocarcinoma ER- cells were more sensitive to Tp-eth extract, showing a decrease in cell viability, with 63% (48 h) and 44% (72 h) in comparison with untreated cells (Figure 2E). Actually, a dose-dependent cytotoxicity of Tp-eth was observed for this cell line.

The Tp-aq extract had a lesser effect on ER- adenocarcinoma cells, with 60% of cells still viable after 72 h of incubation, with the highest concentration of 3.33 mg/mL. For less-concentrated aqueous extracts (0.1 and 0.333 mg/mL), the metabolic activity of cells was not affected.

An increased number of viable cells was observed when Tp-aq and Tp-eth were applied to ER+ adenocarcinoma cells and non-tumorigenic breast cells, (Figure 2A,B) at the lowest concentrations of 0.1 or 0.33 mg/mL, with the values of cell viability exceeding 110% of control samples. This effect can be associated with a pro-proliferative activity of both extracts on ER+ breast cancer cells and, also, on normal mammary gland cells. For MCF-7 cells, a slightly more pronounced pro-proliferative effect was observed for both extracts at the same concentration (Figure 2C) compared with normal breast cells (MCF-12A). This biphasic dose response of *T. pratense* extracts on the proliferation of normal breast and estrogen-dependent adenocarcinoma cells was not reported in the scientific literature until now. Several other studies presented results on individual phytoestrogens or phenolic compounds, which are able to sustain and stimulate cell proliferation at lower concentrations and induce cytotoxicity at a higher dose [14].

LDH release test, which is depicted as cytotoxicity in Figure 2, confirmed the MTS assay’s results showing an increase in LDH release for all three cell lines treated with the highest concentration (3.33 mg/mL) of Tp-eth (Figure 2B,D,F, with *p* < 0.001). For ER- cell line, the cytotoxicity (%) was approximately twice as high as what was obtained for untreated cells (the control sample). Actually, the ethanolic extract at the maximum concentration could induce an LDH leakage, which might be associated with losing cellular membrane integrity on all tested cell lines. For the lowest concentrations of Tp-eth (0.1, 0.33 mg/mL), no significant cytotoxicity was detected for both time intervals on all three cell lines.

Interestingly, the Tp-aq at a concentration of 3.33 mg/mL was able to induce cytotoxicity on both tumorigenic cells (ER+ and ER−) but not on non-tumorigenic cell lines. In fact, we could not detect any significant LDH release on normal gland mammary cells incubated with aqueous plant extracts. Based on these results, the *T. pratense* aqueous extract showed a selective cytotoxic activity toward cancer cells as other plant extracts demonstrated [59]. This property can be a criterion in the search for new anticancer drug candidates with cancer cell-selective toxicity and protection toward normal cells.

The cytotoxicity experiments, which combined cell viability testing and LDH release assessment, showed different biological effects of *T. pratense* extracts on cells depending on ER expression and malignancy status. The most relevant observation, which has not been reported in the literature until now, is that both ethanolic and aqueous extracts induced a biphasic dose response on the cellular proliferation process of ER+ adenocarcinoma and breast normal cells. Additionally, the dose- and time-dependent cytotoxic effect of both extracts on ER- invasive adenocarcinoma cells were observed.

### 3.5. Real-Time Cellular Proliferation Monitoring of Breast Cell Lines Treated with T. pratense Extracts

**Figure 3:**
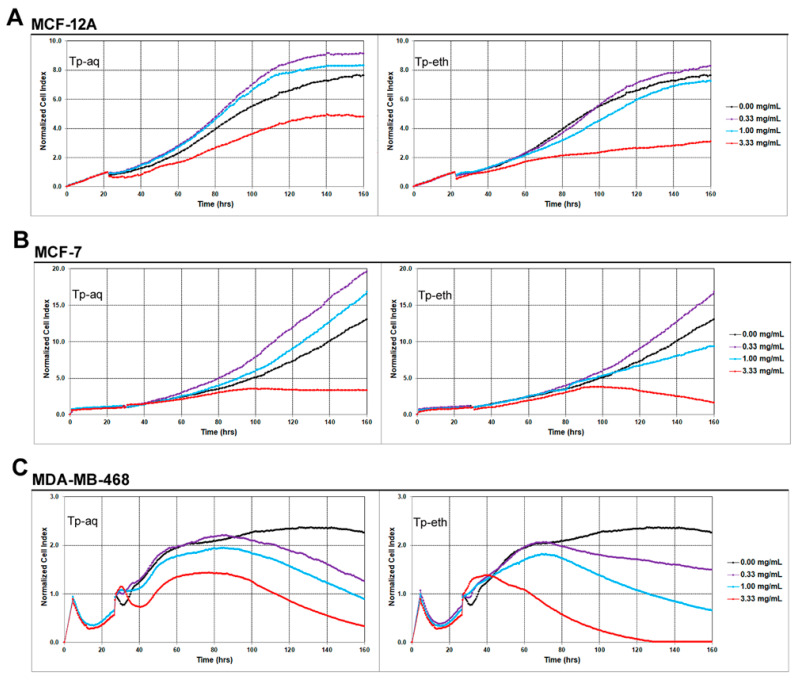
Representative cellular proliferation curves recorded in real time by xCELLigence (RTCA) system: (**A**) MCF-12A cells, (**B**) MCF-7 cells, and (**C**) MDA-MB-468 cells. The cells are untreated (control, 0.0 mg/mL) or treated with indicated concentrations of Tp-aq or Tp-eth (0.33, 1.0, 3.33 mg/mL). Normalized cell index of three different experiments.

Based on the results obtained on viability and cytotoxicity tests, we decided to perform real-time experiments to monitor cell growth for a longer period of time using the xCELLigence system. The RTCA system is continuously recording the cellular impedance, which produces specific time/dose-dependent cell response profiles upon treatment with bioactive compounds. The measurements through many time points enable us to create profiles that reflect cellular proliferation, growth, and morphological modifications before and after adding a compound. Recorded cellular curves can be associated with the modulation of cell physiology by bioactive compound treatment [60]. The cells were observed for 144 h under the treatment with three different concentrations of extracts, namely, 0.33, 1.0, and 3.33 mg/mL, and compared with untreated cells. The representative cell response profiles of all three cell lines incubated with plant extracts are depicted in Figure 3. The addition of Tp-aq at 0.33 and 1.0 mg/mL concentrations to MCF-12A cells induced an increase in CI values above the values obtained for control samples (Figure 3A). This might indicate that the Tp-aq treatment is able to stimulate the cell proliferation rate and to shorten the doubling time of the cell population. An antiproliferative effect was observed at the highest concentration of 3.33 mg/mL, with CI values reaching half the value obtained for the control sample by the end of the incubation period. As for Tp-eth treatment, the cell proliferation curve showed a slight increase for the lowest concentration (0.33 mg/mL) at the last 72 h of treatment and a slight decrease in CI values for the next concentration of 1.0 mg/mL in comparison with the control sample. The results are similar to what was obtained by viability and cytotoxicity test, indicating that the ethanolic extract does not significantly influence the proliferation rate as Tp-aq does, nor does it induce cytotoxicity at 0.33 and 1.0 mg/mL concentrations. Instead, the highest dose of Tp-eth of 3.33 mg/mL produced a negative effect reflected by the cell proliferation profile, which indicates a much stronger antiproliferative effect than what was obtained for Tp-aq treatment. The CI values recorded for Tp-eth at the highest concentration were 1.5 times as low as values obtained for Tp-aq during the last two days of treatment.

A similar effect of Tp-aq on ER+ adenocarcinoma cells was observed (Figure 3B) with increasing CI values at 0.33 and 1.0 mg/mL concentrations, while at the highest concentration of 3.33 mg/mL, a steady level of CI with values below what was registered for control samples was observed. For MCF-7 cells treated with Tp-eth at 0.33 mg/mL and 3.33 mg/mL, the proliferation curves showed a similar pattern to that obtained for Tp-aq at the same concentrations. For treatment with Tp-eth at 1.0 mg/mL the MCF-7 cell response profile mimicked the control cellular proliferation curve during the first 72 h of treatment, whereas for the remaining hours, the CI values decreased showing an antiproliferative profile.

The ER-adenocarcinoma cells were the most affected by the treatment with Tp-aq and Tp-eth, as Figure 3C shows. The antiproliferative effect of both extracts was time- and dose-dependent, with continuously decreasing values of CI in comparison with untreated cells, except for the cells treated with 0.33 mg/mL of extracts, which showed lower CI values for the last 4 days of treatment. However, the CI registered after 72 h of treatment with Tp-eth at a concentration of 3.33 mg/mL reached the values of 0.01–0.1. A significant change in cell proliferation curves was observed immediately after adding the high dose of plant extracts, in comparison with the profiles of cells treated with low doses. This pattern reflects a dynamic cell response curve typical for the induction of cell apoptosis following the addition of extracts, which reflects a transient morphological change, followed by a gradual, dose-dependent decrease in CI [60]. For example, on A549 lung carcinoma cells, the *R. C. stauntonii* extract produced similar time- and dose-dependent cell response profiles; by TUNEL DNA fragmentation assay, the authors confirmed that apoptosis occurred under higher doses of plant extract treatment [60].

In general, the real-time cellular proliferation monitoring data were comparable with the results from viability and cytotoxicity assays. The main observations are that Tp-eth is more cytotoxic and has a significant antiproliferative effect on the ER- adenocarcinoma cell line at all tested concentration in comparison with Tp-aq. Additionally, the biphasic effect induced by both extracts on ER+ adenocarcinoma and on non-tumorigenic mammary gland cells, with a pro-proliferative activity at lower concentrations and an antiproliferative effect at the highest concentration, was registered in real-time experiments. *T pratense*, especially the aqueous extract, can enhance the proliferative process of breast ER+ adenocarcinoma cells, and the non-tumorigenic ER insensitive cells.

No similar effects of *T. pratense* extracts on cell models, supporting cells proliferation at lower concentrations while inducing negative effects at higher concentrations have been reported until now. The biphasic dose–response of several classes of phytochemicals, especially of phytoestrogens on ER+ cell proliferation were documented in scientific reports [14].

### 3.6. Estrogenic Activity of T. pratense Extracts

The increase in cell proliferation observed at concentrations of 0.1–1.0 mg/mL on MCF-7 and MCF-12A cell lines could be explained by possible estrogenic effects exercised by the *T. pratense* extracts, which are rich in phytoestrogens. To investigate this, the cells were incubated for a long period of time (6 days) with extracts at these low doses, in the presence or absence of an ER antagonist (ICI 182,780) and, then, analyzed based on E-screen assay. This molecule acts as a pure antiestrogen by binding to the ERs, causing disassociation of receptor-associated proteins, impaired receptor dimerization, and increased receptor degradation [38].

**Figure 4:**
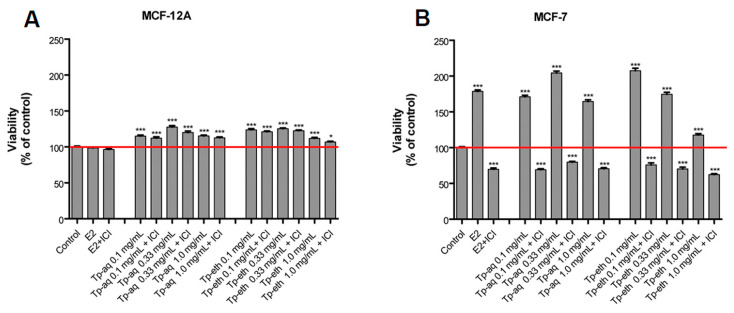
Evaluation of estrogenic activities of Trifolium pratense extracts at different concentrations of 0.1, 0.33, and 1.0 mg/mL on MCF-12A cells (**A**) and MCF-7 cells (**B**) measured by the E-screen assay. The cells treated with 1 nM of E2 represented the positive control and the ICI 182,780 (ICI) solution at 100 nM concentration was used for co-treatment experiments. The red line indicates 100% cell viability. The results represent the mean ± SEM of at least three independent experiments with *p* < 0.05 (*), and *p* < 0.001 (***) compared with the control (untreated cells).

In Figure 4A, the pro-proliferative effect on MCF-12A cells of different concentrations of Tp extracts (0.1, 0.33, and 1.0 mg/mL) can be observed. The proliferation rate for all tested concentrations was statistically significant with *p* < 0.001, in comparison with the control, untreated cells. Interestingly, the same pro-proliferative effect was observed when cells were co-treated with ER antagonist ICI 182,780 at 100 nM, which can indicate a non-estrogenic proliferation process. Moreover, our data indicate that E2 has no ability to facilitate the proliferation of non-tumorigenic cell lines, which is in accordance with previous scientific reports. By using multiple assays, a couple of reports have shown that MCF-12A cells are unresponsive to E2 in culture conditions, both in regard to cell proliferation and the expression of estrogen-regulated genes [61].

The evaluation of the estrogenic potential of *T. pratense* extracts on MCF-7 cells can be observed in Figure 4B. The Tp-eth at 0.1 mg/mL and Tp-aq at 0.33 mg/mL concentration induced strong stimulatory effects, with a proliferation rate close to 200%, being even higher than what was obtained for cells treated with E2 at 1 nM (160%). Our results were in alignment with what was reported by Spagnuolo et al., who found that batches of *T. pratense* with different content of isoflavones have a higher estrogenic potential than E2 and individual isoflavones. They postulate that the phytocomplex composition of extracts can influence the modulation of the pharmacological activity of its individual components [62]. Additionally, for Tp-eth a dose dependency of the proliferative effect was observed, with a maximum of estrogenic effect at a concentration of 0.1 mg/mL; after that, it decreased with the increase in the extract concentration. A plausible explanation is that an increased concentration of extract will provide a higher amount of phytoestrogens. In addition to isoflavones, other flavonoids possess estrogenic activities, and by reaching a threshold of concentration, some of the phytoestrogens might exhibit antagonist effects on ER-positive cells. 

The MCF-7 cell proliferation was mitigated almost completely (60% proliferation rate compared with untreated cells) when cells were co-treated with plant extracts and ICI, confirming that the plant extracts acted through ER signaling pathways.

In conclusion, based on E-screen tests, the *T. pratense* extracts at low doses showed a strong estrogenic effect able to stimulate ER+ adenocarcinoma cell proliferation at a high rate. Instead, the moderate stimulatory effect on proliferation of ER-insensitive normal breast cells could be unrelated to the estrogenic signaling pathways. The MCF-12A cells are considered to mimic the human normal mammary gland cells, so the stimulatory effect on normal cells’ proliferation can be noted as a beneficial effect of *T. pratense* extracts.

### 3.7. Cellular Antioxidant Activity of T. pratense Extracts

The antioxidant activity of extracts was assessed in cells with oxidative stress generated either by ROS or RNS inducers. The MCF-12A and MCF-7 cells were pretreated for several hours (~16 h) with plant extracts with concentrations (0.1–1.0 mg/mL) at which the cellular viability was not affected, as previous experiments showed. Then, the oxidative stress was induced and the cellular response was recorded. The MDA-MB-468 cells’ viability was affected by extracts in a dose-dependent manner; furthermore, the reactive species generators increased the cytotoxicity. Therefore, ER- adenocarcinoma cells did not meet the criteria for testing the cellular antioxidant capacity of extracts.

**Figure 5:**
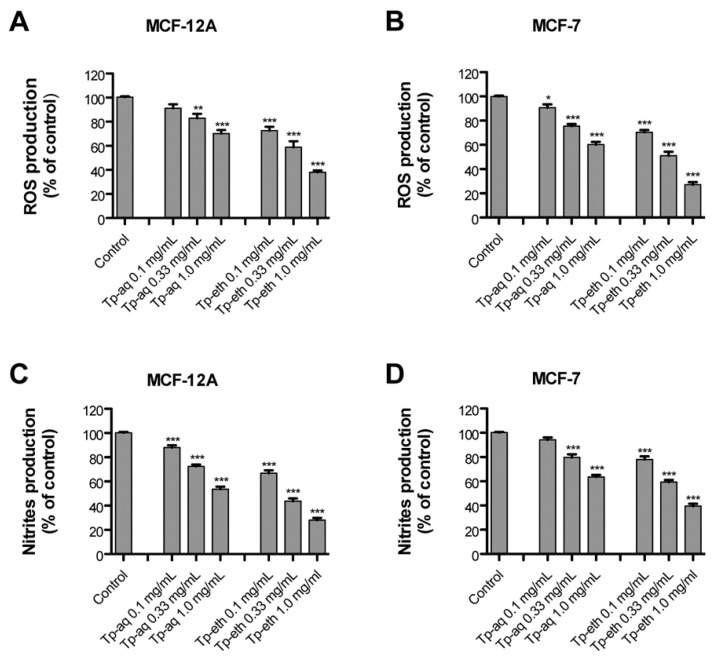
*T. pratense* extracts showed ROS and RNS scavenging potential in MCF-12A and MCF-7 cells. The overnight plant extract-pretreated cells were exposed to free radical initiators for ROS (1 h) and RNS (6 h) generation. ROS production was assessed by cellular antioxidant assay on MCF-12A cells (**A**) and on MCF-7 cells (**B**). Nitrites production was assessed by the Griess assay on MCF-12A cells (**C**) and on MCF-7 cells (**D**). The results of both assays are expressed as a percentage of ROS and nitrates production vs. control (extract untreated cells with induced oxidative stress) and represent the mean ± SEM of at least three independent experiments with *p* < 0.05 (*), *p* < 0.01 (**), and *p* < 0.001 (***).

Figure 5 presents the assessment of the cellular antioxidant capacity of extracts at concentrations of 0.1, 0.33, and 1.0 mg/mL against ROS and RNS. The overnight pretreated cells were exposed to the ROS-initiator compound and the oxidized fluorescent dichlorofluorescein (DCF) signal was recorded (Figure 5A). The decrease in cellular fluorescence of pretreated cells is proportional to the cellular antioxidant capacity of extracts, and it was reported to be the high fluorescence level generated by ROS in cells with induced oxidative stress (control).

The ROS production decreased, in a dose-dependent manner, in MCF-12A cells preincubated with either Tp-aq or Tp-eth extracts, achieving the highest reduction of approximately 60% of fluorescent signal for the Tp-eth at 1.0 mg/mL concentration compared with the control cells (with *p* < 0.001). The same concentration-dependent capacity to scavenge ROS was observed in ER+ adenocarcinoma cells where extracts showed even a higher antioxidant potential at all tested concentrations in comparison with non-tumorigenic cells. The ethanolic extract, which contains a greater quantity of flavonoids than the aqueous one, acted as a more promising antioxidant source in this experimental setting.

Nitric oxide is a free radical produced in mammalian cells that is involved in the regulation of various physiological processes. However, excess production of NO is associated with the inflammation process, which is a precondition in many chronic diseases, including breast cancer [63]. Nitric oxide is a very unstable molecule that reacts with O_2_ to produce stable nitrate and nitrite species. The nitrites production can be assessed using the Griess reagent, and the RNS scavenger capacity of a plant extract can be estimated in cells under externally induced oxidative stress. In the present study, the nitrite produced by the incubation of cells with the SNP solution was significantly reduced by both extracts. This may be due to the antioxidant capacities of flavonoids found in extracts, which compete with oxygen to react with nitric oxide, leading to the reduced production of nitric oxide.

Pretreatment of cells with *T. pratense* extracts at indicated concentrations (0.1, 0.33, and 1.0 mg/mL) reduced, in a dose-dependent mode, the nitrite level generated by SNP in both types of cells, as Figure 5B shows. Overall, a higher reduction was observed for non-tumorigenic cells (MCF-12A) in comparison with ER+ adenocarcinoma cells (MCF-7) for all tested concentrations. Nevertheless, the ethanolic extract possesses a greater RNS scavenging capacity in both cells as the experimental data showed. At the highest concentration of 1.0 mg/mL, the Tp-eth reduced the nitrites production by close to 28% versus 55% when Tp-aq was applied to MCF-12A cells. In MCF-7 cells, the Tp-eth (60%) was two times more potent in reducing the RNS generated by SNP than the Tp-aq (30%). However, for MCF-7 cells pretreated with 0.1 mg/mL of Tp-aq, the nitrite concentration remained unchanged compared with the control, yet for a higher concentration of Tp-aq (0.33–1.0 mg/mL), the RNS reduction was robust, statistically relevant with *p* < 0.001.

In summary, the aqueous and ethanolic extracts at concentrations ranging between 0.1 to 1.0 mg/mL showed a concentration-dependent scavenging potential to inhibit the excess of radical species in normal and adenocarcinoma ER+ breast cells under oxidative stress.

### 3.8. Pro-Oxidant Effect of T. pratense Extracts

Plant extracts contain polyphenols, which can have antagonist activities, both anti- and pro-oxidant effects in cellular systems. Over the past decade, numerous studies have shown that polyphenolic compounds have different redox potentials that can modulate antagonist effects on cells [10,64]. As antioxidants, they scavenge intracellular ROS, and conversely, they can act as pro-oxidant agents by generating ROS under certain conditions, such as high doses or in the presence of transition metals [64]. Therefore, by producing oxidative stress the plant polyphenols can induce DNA damage and lipid or protein oxidation, which can promote cell death [7]. In addition, several studies reported that antiproliferative activity and cytotoxic effect of polyphenolic compounds may be mediated by their capacity to induce oxidative stress by generating intracellular ROS [8].

**Figure 6:**
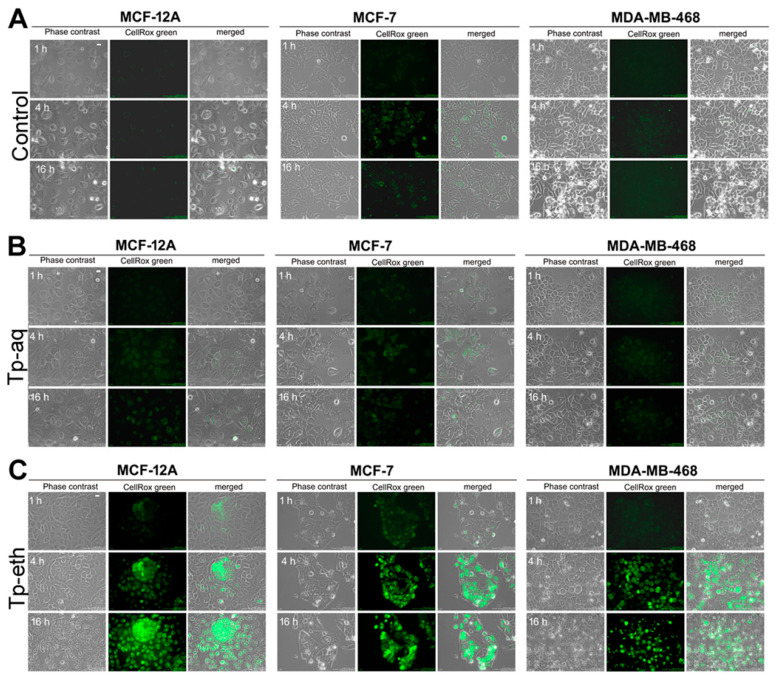
The detection of ROS generation using CellROX green reagent. The cells previously loaded with CellROX were left untreated for control (**A**), treated with 3.33 mg/mL concentration of Tp-aq extract (**B**) and Tp-eth extract (**C**), and observed under the microscope for 24 h. The representative images obtained at 1, 4, and 16 h after starting the incubation of MCF-12A, MCF-7, and MDA-MB-468 cells with extracts are presented. The live cell imaging was performed using a BioStation IM platform (Nikon); the time-lapse images were taken with 20× objective on phase contrast and green channel. Scale bar = 10 μm.

Based on real-time cellular proliferation profiles, which showed a strong antiproliferative effect on MCF-12A and MCF-7 cells and cell death induction on MDA-MB-468 at high doses of extracts (3.33 mg/mL), the possibility of intracellular ROS generation was investigated using time-lapse imaging microscopy. The previously CellROX green-stained cells were treated with plant extracts at two concentrations (1.0 or 3.33 mg/mL) and were immediately placed into the BioStation IM platform and monitored for 24 h. The CellROX green reagent becomes fluorescent only upon oxidation by ROS and then binds to DNA, showing a signal localized primarily in the nucleus and mitochondria. Therefore, the increase in the fluorescent signal in the cells will be an indicator of greater ROS production. The representative images captured at 1, 4, and 16 h after cell treatment started and the images of untreated cells at the same interval of time are presented in Figure 6. No significant increase in fluorescent signal for the observed period of time can be detected for untreated cells (control) (Figure 6A). Both tumor cell lines showed a basic weak fluorescent signal and a lack of measurable fluorescent signal for normal mammary gland cells. Indeed, tumor cells present a basal production of ROS, which is a consequence of increased rates of metabolism and a defective antioxidant system compared with non-tumorigenic cells [65].

The experimental settings allowed us to monitor four samples simultaneously and record images in each selected field. So, the signal generated by ROS within Tp-eth-treated cells was recorded simultaneously with a very weak or a lack of fluorescent signal within untreated cells. At a 4-h time point, strong fluorescence localized in the nucleus and mitochondria was captured for all types of cells treated with the highest concentration of Tp-eth (3.33 mg/mL), and the signal maintained its intensity for all monitored periods of time (Figure 6C). No such intense fluorescence can be observed within cells incubated with the same concentration of Tp-aq extract (Figure 6B), although the ROS production cannot be excluded. Weak green fluorescence was noticed in all types of cells treated with aqueous extract when visually compared with corresponding untreated cells.

The production of ROS was also observed when the cells were treated with Tp-eth at 1.0 mg/mL concentration (Figure S1) although the signal was dimmer in comparison with the cells exposed to the more concentrated ethanolic extract. However, the adenocarcinoma ER− cells displayed the strongest signal compared with adenocarcinoma ER+ and ER− non-tumorigenic cells exposed to the same concentration of extracts. Therefore, by monitoring the intracellular ROS generation using fluorescent time-lapse microscopy, strong dose-dependent oxidative stress can be observed for cells treated with Tp-eth extract, in comparison with cells treated with Tp-aq extract.

### 3.9. Mitochondria and ATP Levels Evaluation

To further explore the intracellular oxidative stress occurrence in cells incubated with high dose of plant extract, the ATP production and the mitochondria activity were investigated. Mitochondria plays a crucial role in cellular energy production, and its activity can be compromised by ROS overproduction with profound implications for cell homeostasis. Extra generation of ROS can damage mitochondrial permeability and structure and might have an impact on ATP synthesis in cells [7]. Consequently, the ATP level becomes a key indicator of mitochondrial health and the metabolic state of cells. A bioluminescence assay was used to measure the mitochondrial ATP production of cells treated for 48 h with low and high doses (0.33 and 3.33 mg/mL) of extracts. This ATP assay accurately detects the viability and metabolic activity of cells, has a higher sensitivity, and is less prone to artifacts than other cell viability assays based on colorimetric or fluorometric measurements.

**Figure 7:**
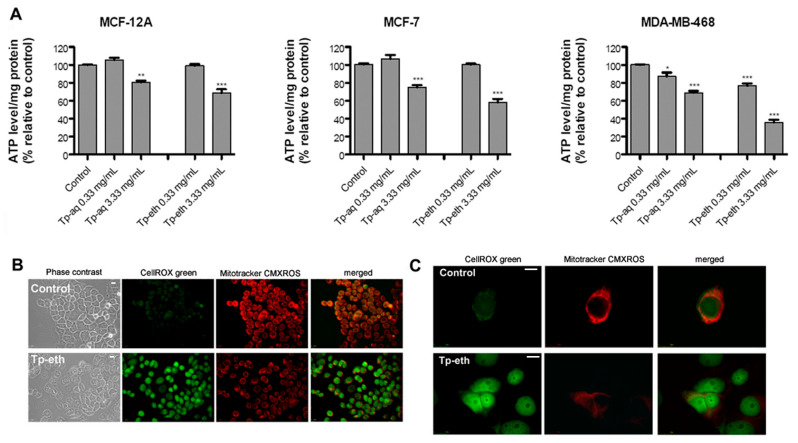
The ATP level reported for the protein concentration and expressed as % relative to the control (untreated cells) treated for 48 h with Tp extracts at concentrations of 0.33 mg/mL and 3.33 mg/mL. Each experiment was repeated three times and the results represent the mean ± SEM with *p* < 0.05 (*), *p* < 0.01 (**), and *p* < 0.001 (***) compared with the control (**A**). Representative images of MDA-MB-468 cells double-stained with CellROX green for ROS and MitoTracker Red CMXRo for active mitochondria after 48 h of Tp eth (3.33 mg/mL) treatment or untreated cells (control). Images collected with 20× objective (**B**) and 80× objective (**C**) of the Eclipse TE300 Nikon microscope. Scale bar, 10 μm.

As Figure 7A shows, the ATP level decreased in all three cell lines treated with the highest dose of Tp-eth (3.33 mg/mL). The most significant depletion of ATP was observed in ER- adenocarcinoma cells, MDA-MB-468, which had close to 60% less ATP content compared with the control. The ER+ adenocarcinoma (MCF-7) cells showed 40% and non-tumorigenic, MCF-12A, cells almost 30% less ATP production in comparison with the corresponding controls. The same extract at low dose (0.33 mg/mL) in MCF-12A and MCF-7 cells alleviated such energy inhibition, which was consistent with the results from cell viability and real-time cellular analysis experiments. Instead, for MDA-MB-468 cells, a statistically significant (*p* < 0.001) depletion of ATP production was observed for Tp-eth at 0.33 mg/mL. Both tumorigenic cells, MDA-MB-468 and MCF-7, exposed to the highest concentration of 3.33 mg/mL of Tp-aq lost more than 30% and 25% of their ATP levels, respectively. These changes were statistically significant, with *p* < 0.001 when compared to control. The non-tumorigenic cells were less affected by this treatment, with an approximately 20% (with *p* < 0.01) decrease in ATP level in comparison with control cells. No significant modifications of ATP metabolism were observed for Tp-aq-treated cells at 0.33 mg/mL with the exception of MDA-MB-468 cells, which had approximately 12% less ATP in comparison with the control.

The effect of high-dose extracts on ATP production might be a sign of mitochondria damage induced by the generation of ROS. Next, the presence of oxidative stress after 48 h of incubation with high-dose plant extracts was evaluated. The ROS were stained with CellROX green reagent and mitochondria with MitoTracker^®^ red CMXRos; then, the cells were fixed in 4% paraformaldehyde and observed under the fluorescent microscope. The Mitotracker is a cell-permeable reagent and is non-fluorescent until it binds covalently to mitochondrial lipids and proteins. The red fluorescent dye accumulates in active mitochondria, exhibits good photostability, and responds to changes in mitochondrial membrane potential [65]. In addition, both dyes are retained after cell fixation, so the oxidative stress and the effect on mitochondrial potential can be observed simultaneously after exposure to the highest dose of extracts (3.33 mg/mL). A decrease in the intensity of MitoTracker reagent staining indicates an alteration in mitochondrial activity, and the increase in green fluorescence in the nucleus and mitochondria is signaling the overproduction of ROS.

The representative images of double-stained MDA-MB-468 cells treated with 3.33 mg/mL of Tp-eth extract and the untreated cells (control) can be observed in Figure 7B. The population of untreated cells largely resembles healthy and proliferating cells with intact mitochondria as the bright red fluorescence shows. The dimmer CellROX green signal indicates a very low presence of ROS. In comparison, a large number of cells exposed to Tp-eth extract presented a strong CellROX signal localized in the nucleus, signalizing ROS generation even after 48 h of treatment. A relatively weaker red fluorescence was present in the majority of treated cells, compared with the untreated cell population. Thus, the double-staining method revealed that by inducing oxidative stress through ROS generation, the mitochondria potential of treated cells can be affected. Examples of treated and untreated MDA-MB-468 cells observed at higher magnification can be found in Figure 7C. The other two cell lines were tested in the same condition and a smaller population of cells presented the same pattern of staining after treatment with Tp-eth at a concentration of 3.33 mg/mL, consistent with ATP assay results. 

No significant differences were observed between the intensity of the Mitotracker signal for all three cell lines incubated with Tp-aq at 3.33 mg/mL and the corresponding controls. The ROS signal was weaker, similar to what was observed for 24 h treatment experiments, but a strong staining of mitochondria was detected, similar to the staining of untreated cells (unpublished data).

The obtained data suggest that high-dose Tp-eth extract has a greater capacity to reduce the mitochondrial membrane potential and ATP production than Tp-aq extract in 48 h.

### 3.10. Evaluation of Lipid Peroxidation

**Figure 8:**
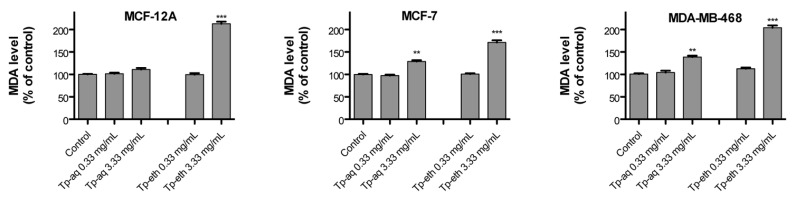
MDA content measured in cells exposed to different concentrations of plant extracts for 48 h. The results represent the mean ± SEM of at least three independent experiments with *p* < 0.01 (**), and *p* < 0.001 (***) compared with the control (untreated cells).

Next, we analyzed whether the pro-oxidant activity of high-dose extracts impacted the cellular oxidative stress markers. Lipid peroxidation is one of the indicators of oxidative damage and implies that ROS mediate the degradation of polyunsaturated fatty acids localized in cell membranes [7]. The by-products of lipid peroxidation, such as malondialdehyde, can be detected by TBAR assay. The cells were exposed for 48 h to 0.33 and 3.33 mg/mL concentrations of extracts and then were analyzed, with the results presented as MDA content reported to the control (Figure 8).

The MDA content was two times as high in MDA-MB-468 and MCF-12A cells treated with Tp-eth at the 3.33 mg/mL concentration as in the untreated cells. For MCF-7 cells, the MDA level increased 1.75 times compared with what was obtained for the control sample. In the case of Tp-aq treatment at the same concentration, lipid peroxidation was present for the two tumorigenic cell lines (MCF-7 and MDA-MB-468) with a significant statistic (*p* < 0.01) increases in MDA content in comparison with untreated cells. The results obtained at lower concentrations were close to what was obtained for the control, and no lipid peroxidation could be observed for cells treated for 48 h with plant extracts at a concentration of 0.33 mg/mL.

The obtained results confirm that the high concentration of Tp-eth extract can affect lipid peroxidation, while at the same concentration of Tp-aq, a slight increase in the MDA content was determined just for the two tumorigenic cell lines.

### 3.11. SOD Activity and GSH Level Evaluation

For counteracting the ROS damaging effects, cells use antioxidant defense systems formed by enzymes such as SOD or by endogenous antioxidant molecules such as GSH. The SOD enzymes have a direct role in catalyzing the dismutation of superoxide anion (O^2−)^ into hydrogen peroxide and molecular oxygen. In addition, SODs also participate in cell signaling processes through the H_2_O_2_ molecules, which can act as a second messenger to modulate inflammation, angiogenesis, and gene expression [3].

The GSH is an antioxidant molecule necessary for reducing ROS and thiol within cells and responsible for its cellular availability is glutathione reductase, part of the enzymatic antioxidant defense system. The reduced glutathione is a tripeptide thiol (γ-glutamyl cysteinyl glycine) molecule and its intracellular concentration is an indicator of oxidative stress. Within the cells, glutathione in reduced form can neutralize ROS, including superoxide anion (O_2_^–^), hydroxyl radical (·OH), hydrogen peroxide (H_2_O_2_), or other organic radicals through a concerted cascade of detoxification mechanisms. A depletion of GSH in cells can compromise the intracellular redox homeostasis leading to alterations in the cellular redox balance, which can disrupt redox signaling events that modulate cell death activation and progression [66].

**Figure 9:**
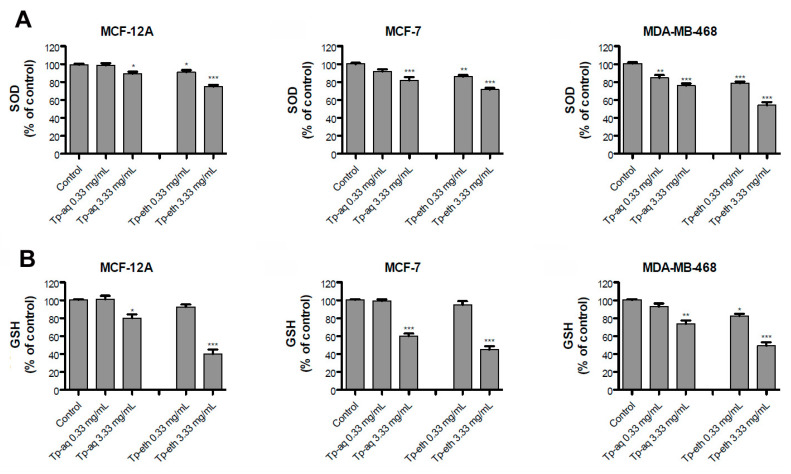
The effect of Tp extracts at 0.33 and 3.33 mg/mL concentrations on the SOD activity (**A**) and GSH levels (**B**) in cells treated for 48 h. The results represent the mean ± SEM of at least three independent experiments, with *p* < 0.05 (*), *p* < 0.01 (**), and *p* < 0.001 (***) as compared with control (untreated cells).

The activity of SOD and GSH levels were investigated in cells incubated for 48 h with the 0.33 mg/mL and 3.33 mg/mL concentrations of plant extracts and the results are presented in Figure 9. All cells treated with Tp-eth at 3.33 mg/mL showed a significant downregulation of the SOD activity (Figure 9A), with the most reduction of 50% observed in ER- adenocarcinoma cells (MDA-MB-468). The low dose of Tp-eth (0.33 mg/mL) affected the SOD activity in all tested cells. The most significant decrease was observed in MDA-MB-468 cells, with almost 20% of reduction in comparison with the control (with *p* < 0.001), followed by MCF-7 (15%, with *p* < 0.01) and MCF-12A cells (12%, with *p* < 0.05).

The high-dose Tp-aq extract led to a less pronounced decrease in the SOD activity than Tp-eth but still statistically significant in comparison with control samples, with *p* < 0.001 for both adenocarcinoma cells MCF-7 and MDA-MB-468 cells, and with *p* < 0.05 for non-tumorigenic MCF-12A cells.

Nevertheless, the ER- adenocarcinoma cell line (MDA-MB-468) was the most affected in terms of the SOD activity by both extracts at all tested concentrations.

A strong depletion of GSH levels was observed in all cell lines when the high concentration of Tp-eth was applied for 48 h, as Figure 9B shows. The GSH level decreased significantly in MCF-12A cells by almost 60%, followed by 55% for MCF-7 cells, and 51% for MDA-MB-468 cells, compared with the corresponding controls. In the case of MCF-12A and MCF-7 cells, the reduction in the GSH level was even more pronounced than the decrease in the SOD activity. A moderate effect on the GSH level was observed when the high-dose Tp-aq was applied to all three cell lines, with a depletion close to 40% for MCF-7 cells, followed by almost 30% reduction for MDA-MB-468 cells, and, finally, approximately 20% reduction for MCF-12A in comparison with the corresponding controls. The low doses of both extracts did not affect the GSH level, with the exception of Tp-eth extract, which reduced the GSH level to 82% (with *p* < 0.05) in MDA-MB-468 cells compared with untreated cells.

Overall, the Tp-eth at a high dose had a significant effect on the SOD activity and GSH content in all breast cell lines.

### 3.12. Apoptosis Assessment

**Figure 10:**
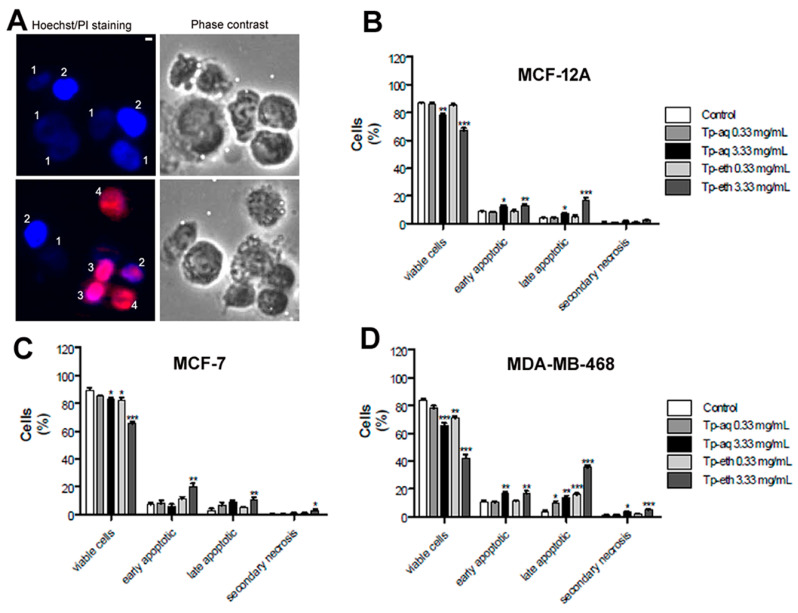
**Apoptosis assessment.** (**A**) Representative composite images showing the morphological changes of treated cells with the 3.33 mg/mL Tp-eth extract, detected with dual staining of Hoechst 33342/PI. Cells were treated with Tp extracts for 48 h and imaged by fluorescence microscope and in phase contrast (magnification 20×). Scale bar, 10 μm. Numbers indicate (1) viable cells with normal nuclei: Hoechst dim/+; (2) early apoptotic cells: Hoechst++/PI− or +; (3) late apoptotic cells: Hoechst++/PI++ and (4) dead cells with secondary necrosis: Hoechst−/PI++. Percentage of cells classified as viable, early apoptotic, late apoptotic, and secondary necrosis for MCF-12A (**B**), MCF-7 (**C**), and MDA-MB-468 (**D**). Each experiment was repeated three times and the results represent the mean ± SEM with *p* < 0.05 (*), *p* < 0.01 (**), and *p* < 0.001 (***) compared with the control.

To further explore the cytotoxic effect of high-dose plant extracts, experiments to detect apoptosis by assessing cellular morphological changes were conducted. The 48 h incubation of cells with extracts at low and high concentrations (0.33 and 3.33 mg/mL) was followed by double-staining with Hoechst 33342 and propidium iodide (PI) solutions. Thus, the stained cells can be visualized using a fluorescent microscope, and their status regarding viability and apoptosis stages can be quantified. The apoptosis process features typical well-defined morphological changes, including chromatin condensation and DNA fragmentation, nuclear convolution, the formation of apoptotic blebs within the plasma membrane, and a reduction in cell size [40]. These changes can be observed, identified, and classified as early and late apoptosis events. The blue fluorescent Hoechst 33342 is a cell-permeable nucleic acid binding dye; whereas, the red fluorescent PI is a membrane-impermeable reagent, so it can only enter cells with compromised membranes. PI and Hoechst 33342 double-staining allow for differentiating between viable and dead cells [40]. The results obtained in apoptosis assessment experiments are presented in Figure 10.

The classification of cells was based on their staining intensity and was correlated with morphological changes observed in phase contrast, accordingly (Figure 10A). Thus, the absence of PI staining and a weak signal of the Hoechst 33342 reagent are specific for viable cells, which have normal-size nuclei and intact cellular membranes. Instead, a strong blue signal and a lack of partial PI staining are specific for condensed chromatin of shrinking cells and can be attributed to early apoptotic cells. The late apoptotic cells presented an overlap of both staining, with strong signals due to leaking cellular membranes and further DNA condensation. Stage four is represented by secondary necrotic cells characterized by a lack of Hoechst 33342 staining and a strong PI signal when apoptotic cells are not efficiently cleared by phagocytes, leading to the progression of apoptosis into a necrotic state [40].

The experiments confirmed the cytotoxic activity of the high-dose plant extracts on all types of cells. The results showed a decline in the MDA-MB-468 cell population, which had 42% viable cells and 35% late apoptotic cells after being treated with the Tp-eth extract at 3.33 mg/mL (Figure 10D). In comparison, the control showed 84% of viable cells and approximately 5% of late apoptotic cells. A high dose of Tp-eth induced a decrease in viable cells on MCF-7 (Figure 10C) and MCF-12 cells (Figure 10B) and, consequently, an increase in early and late apoptotic cells when compared with the corresponding controls. However, the extent of the percentage of apoptotic cells was not the same as for MDA-MB-468 cells. The Tp-aq treatment at a 3.33 mg/mL concentration produced more damage to MDA-MB-468 cells than to the other two cell lines. After 48 h of incubation with a lower concentration of extracts (0.33 mg/mL), the MDA-MB-468 cell viability was the most affected in comparison with untreated cells. The MCF-7 and MCF-12A cell populations were less affected by the lowest concentration of both extracts, with a smaller percentage of apoptotic cells and with viable cells at a percentage above 80%, as can be seen in Figure 10C,D.

## 4. Discussion

Dietary plant-based supplements, including red clover, are becoming increasingly popular worldwide as they are used to prevent acute and chronic diseases and improve health conditions. Thus, the scientific community has started to investigate the biological effects of popular herb extracts to explore phytochemical content and their potential pharmacological uses and to establish their efficacy and safety [4]. In the past, the *T. pratense* extracts have been studied mainly to establish their estrogenic activity, yet in recent years, other biological effects, such as cellular antioxidant and anti-inflammatory activities, have been demonstrated [19].

The present study started from the hypothesis that the differences in phytochemical content will dictate the intensity of biological activities of extracts on different breast cell lines. In addition, it aimed to investigate the potential differential response of human breast cells incubated with different doses of extracts and to establish if there is a dependency with cells’ tumorigenic status and ER expression. Thus, using a wide range of concentrations (0.1 to 3.33 mg/mL), the study has analyzed how the extracts modulate cellular processes such as cellular viability, proliferation, and oxidative stress. Human breast cell lines used in this study were as follows: MDA-MB-468, which are highly invasive adenocarcinoma triple-negative cells (ER^−^, PR^−^, Her2^−^); MCF-7, which are noninvasive adenocarcinoma ER positive (ER+) cells; and MCF-12A, which are non-tumorigenic ER-negative (ER−), mimicking normal breast cells.

The cytotoxicity results and real-time cellular proliferation monitoring experiments revealed that both *T. pratense* exercised a biphasic dose effect on adenocarcinoma ER+ and normal ER− breast cells. As MTS tests and the proliferation curves show, at low doses, an increase in cell proliferation was observed, and at high doses, an antiproliferative effect occurred. This biphasic dose response was not found when adenocarcinoma ER− cells were tested. Instead, a dose- and time-dependent decrease in cell viability and proliferation rate was observed, with a significant drop in cell growth profile after 48 h of treatment with both extracts at the highest concentration of 3.33 mg/mL.

The literature has reported that dietary phytoestrogens, at low doses, yet physiologically relevant, were able to stimulate the growth of estrogen-sensitive cells, presumably due to estrogenic agonist activity, and to suppress their proliferation at high levels by blocking DNA synthesis and inducing cell death. These findings have been correlated with their capacity to act as agonist or antagonist ligands on ERs for modulating different cellular processes [14].

The mechanisms of action ascribed to phytoestrogens are based on their genomic and non-genomic ER interactions, along with their ER-independent activities, for instance, as epigenetic modulators. The genomic mechanism implies a direct binding to ERs, followed by nuclear translocation and interaction with estrogen response elements (EREs) or with non-ERE sites from gene promoters. Finally, the agonist activity relies on the activation of ER-dependent gene transcription that controls cellular processes like cell growth, differentiation, and cell survival [13,67]. Alternatively, estrogenic molecules can induce rapid non-genomic effects mediated by plasma membrane-associated ERs (mERα and mERβ) or by the intracellular transmembrane G protein-coupled estrogen receptor (GPER) [13,67]. Thus, complex intracellular events are activated, such as cytoplasmatic kinase cascades, ion channels efflux, and interaction with growth factor receptors, leading to the modulation of various gene expressions.

Recent emerging evidence highlighted the phytoestrogens’ capacity to act through ER-independent pathways by modulating epigenetic mechanisms [6,13,67]. DNA methylation, histone post-translational modifications, and non-coding RNA expression are the main epigenetic mechanisms that regulate gene expression [67,68]. Through a dynamic mechanism, the active epigenetic markers on DNA sequences and histones are produced to signal active or inactive transcriptional sites. Many chronic diseases, including cancer, might be generated by early epigenetic alteration that can be reversed by phytochemicals [5,68,69]. Isoflavones can directly bind and inhibit the DNMT and HDAC proteins, which are responsible for DNA methylation and histone deacetylation in epigenetic mechanisms. So, isoflavones can modulate global and local DNA methylation patterns and, consequently, can influence the expression of genes involved in cellular processes [5,6,67]. Genistein modulates the levels of global DNA methylation and DNA methyltransferase (DNMT) activity in ER-positive and ER-negative breast cancer cells. It has the ability to demethylate and activate various silenced tumor suppressor genes, like ataxia telangiectasia mutated (ATM), phosphatase and tensin homolog (PTEN), and mammary serpin peptidase inhibitor (SERPINB5) by direct binding to the DNMT1 catalytic region [69]. Biochanin A at physiological concentrations modulates histone methylation patterns by inhibiting the activity of lysine-specific histone demethylase 1A (LSD1) in gastric cancer cells [70]. Moreover, on ER-positive cells, biochanin A and formononetin can accelerate cell proliferation by upregulating miR-375 expression and, consequently, activate the ERα-miR375-PTEN-ERK1/2-bcl-2 pathway through an ER-independent mechanism [6]. Furthermore, in vivo studies on diabetic rats have demonstrated that long-term formononetin treatment could reduce oxidative stress and improve insulin resistance, potentially through re-expression of the epigenetic regulator protein, Sirtuin 1 (SIRT1), in kidney tissues [71].

More likely, there is a cross-talk between the genomic and non-genomic actions and epigenetic modulator activity of estrogenic molecules, allowing them to fine-tune the expression of various genes, thus affecting several cellular processes in cancer and normal cells depending on or regardless of ER status.

To further investigate whether the stimulatory proliferation effect of extracts was mediated through estrogen-depending pathways, the E-screen assay was performed on MCF-7 and MCF-12A. The results showed that by long-term incubation of MCF-7 cells with low doses of extracts, a stimulatory effect on cell proliferation was induced. Moreover, the rate of cell proliferation was even higher than what was observed for E2 alone. The co-treatment of cells with extracts and ER antagonist (ICI 182,780) inhibited the ER+ cell’s growth, and the rate of proliferation was downregulated for all tested conditions to approximately 60% compared with untreated cells. Our results were in alignment with what was observed in other studies regarding the agonistic effect of plant extracts rich in phytoestrogens. Despite the fact that phytoestrogens have a low affinity to both ERs, with a greater binding preference for ERβ compared with ERα, the ERs-mediated gene transcription levels can sometimes be higher than those induced by E2 [13]. The two nuclear ERs have opposite functions, with ERα participating in cell proliferation and survival, while ERβ modulates ERα activity when both are co-expressed in cells and tissues. Thus, ERβ activation might reduce proliferation-related transcriptional activity of ERα. Therefore, based on the higher affinity of phytoestrogens for ERβ, it should be reasonable to assume that at physiological concentrations, they will not be able to activate ERα. Yet, several studies have demonstrated that plant extracts with estrogenic activity have the capacity to activate several ER-mediated signaling pathways with higher potency than isolated phytoestrogens or E2. A red clover extract at a concentration of 20 μg/mL containing a high amount of hydrolyzed isoflavones (30%) showed an increased potency to activate ERE- mediated transcription, stronger than E2 or single isoflavone in MCF-7 and Ishikawa cells. Moreover, the extract increased progesterone receptor (PR) and alkaline phosphatase (AP) mRNA expression at higher levels than biochanin A and genistein alone [72]. A study on red clover extracts with different isoflavone content demonstrated that the estrogenic proliferative capacity depends on their ratios of 5,7-dihydroxyisoflavones versus 7-hydroxyisoflavones. In addition, a mixture of the major isoflavones (biochanin A, formononetin, genistein, and daidzein) at the highest concentration found in an extract was not able to induce the same proliferative estrogenic efficacy as the extract itself [62].

In the current study, the *T. pratense* extracts acted, at low doses, as agonists by activating ER-cellular proliferation pathways in breast adenocarcinoma ER-positive cells. Based on the results of isoflavone quantification, both extracts showed a moderate content, yet they exerted a potent estrogenic effect based on E-screen results. This can be explained by the presence of other unquantified phytoestrogens, such as coumestans, lignans, and approximately 40 different isoflavones (e.g., prunetin, irilone) that can act additionally with the four quantified isoflavones to induce a strong estrogenic effect of the whole plant extract [19].

In normal breast cells, the ER antagonist (ICI 182,780) had no effect on plant extract-induced cell proliferation. The rate of proliferation remained higher than in the control, with values close to those obtained for plant extract-treated cells in the absence of ER antagonists. Thus, the results might suggest an ER-independent pathway for the pro-proliferative effect of extracts on normal breast cells. This is a reasonable assumption knowing that the MCF-12A cells are described in the literature as breast cells lacking ERs or expressing extremely weak ER transcripts [61].

No study of the biological effects induced by *T. pratense* extracts on normal breast cells has been reported until now. Instead, individual phytoestrogens at physiological concentrations have been reported to modulate different cellular processes through ER-independent mechanisms. Genistein and quercetin stimulated the proliferation of other normal breast cells that do not express ERs (MCF-10A cells) and the effect was not abolished by ICI 182,780 or by E2 co-treatment [73]. Moon et al. tested genistein and biochanin A on MCF-12A cells at 1 μM concentration and observed beneficial effects on genes involved in breast cell proliferation (e.g., tumor suppressor neurofibromin 2) mediated by independent ER mechanism [74]. Another study indicated that genistein epigenetically regulated the expression of tumor suppressor genes p21 and p16 by influencing histone post-translational modifications and, consequently, inhibited the recruitment of oncogenic BMI1-c-MYC complex during early breast tumorigenesis in vitro and in vivo [6]. Recently, stilbenes were reported to have the ability to produce subtle alterations of DNA methylation patterns in regions of transcriptional regulation (e.g., promoters and enhancers) of several genes related to cancer progression, aging, and metabolism disorders. The incubation of MCF-10A cells with noncytotoxic doses of resveratrol for a long period of time changed the methylation pattern of several genes. Among them, RUNX3, which influences cell cycle and apoptosis, was found to be epigenetically modulated in normal breast cells and, also, in plasma DNA from healthy rats fed with stilbene-based supplements [75].

Thus, *T. pratense* extracts may promote the proliferation of normal, non-tumorigenic breast cells through an ER-independent mechanism, potentially involving epigenetic processes. However, further research on investigating epigenetic mechanisms and the molecular basis of normal cell proliferation in the presence of phytoestrogens is required.

For decades, the concept of plant extracts’ chemoprevention potential has revolved around the idea that their bioactive components can act as antioxidant agents. A potential antioxidant agent should be able to directly scavenge free radicals or effectively suppress Phase I metabolism reactions to reduce the overall cellular oxidative burden and/or to enhance activities of Phase II metabolism reactions by activating antioxidant enzymes [12].

In this study, the examined *T. pratense* extracts underwent a preliminary evaluation of antioxidant efficacy, which included a screening of their DPPH radical scavenging abilities and reducing power (copper and ferric ions) in inorganic experimental systems. The results showed that the Tp-eth extract has approximately two times as much of antiradical and reducing ability as Tp-aq. These results correlate with their different polyphenolic and flavonoid content and are in accordance with what was previously reported by others [42,46,54]. Interestingly, the total amount of the four quantified isoflavones was similar for both extracts, which may suggest that differences in the antioxidant capacity of extracts is based on the content of other classes of plant polyphenolics rather than on isoflavonoid content. Studies on structure–activity relationships revealed that among others, the position and number of hydroxyl groups on the aromatic rings significantly influence the phytochemical antioxidant capacity. Compounds with multiple hydroxyl groups, particularly in ortho or para positions, tend to exhibit stronger antioxidant properties due to enhanced resonance stabilization and intramolecular hydrogen bonding [53]. Antonescu et al. found that the ethanolic extract of Romanian *T. pratense* was rich in flavonols such as rutin and catechin and phenolic acids such as ferulic and caffeic, all having a strong scavenging activity [42]. Our preliminary HPLC analysis on flavonoid and polyphenol quantification (unpublished data) shows that the Tp-eth contains approximately two times as much rutin and 3-glycosylated quercetin as the Tp-aq extract. Regarding the phenolic acids, the biggest differences were found for caffeic acid, yet similar amounts of others like chlorogenic, trans-ferulic, and rosmarinic acid were determined in both extracts. In general, the isoflavonoids have lower antiradical and reducing capacity than other polyphenolics mentioned above. Moreover, the two 4′-O-methoxy isoflavones (formononetin and biochanin A) are less potent than their precursors, daidzein and genistein [28]. Therefore, it is assumed that flavonoids with multiple hydroxyl groups (especially a catechol group), such as quercetin and kaempferol, along with phenolic acids, which possess dihydroxylation structures like caffeic acid, among others with similar structures, are the principal contributors to scavenger and reducing capacities of *T. pratense* extracts.

Nevertheless, as no single chemical method can predict in vivo effectiveness of the antioxidant capacity of plant extracts, we tested them at doses for which the cells are viable even in oxidative stress-induced conditions. The results show that both extracts have the capacity to scavenge ROS and RNS generated in adenocarcinoma ER+ and in non-tumorigenic ER− cell lines in a concentration-dependent manner. Congruently with other reports, the intracellular ROS scavenging capacity of extracts was significantly higher, especially for Tp-eth. For instance, a 70% ethanolic extract of Romanian red clover showed a potent scavenging activity in normal human fibroblast cell lines (BJ cells) under oxidative stress induced by H_2_O_2_. At concentrations between 0.1 and 0.3 mg/mL, the ROS production in viable BJ cells was reduced with an increase in a concentration of extracts. In addition, the biphasic dose effect on cells proliferation was observed [76]. In non-stimulated conditions and in the presence of a pro-oxidant (t-BOOH), the fermented *T. pratense* sprout extracts decreased the ROS production in breast adenocarcinoma ER-+ and ER− cells and in human umbilical vein endothelial cells to a lesser extent (approximately 20–40%) than what we observed in our study (30–65%) for MCF-7 cells. The biphasic dose effect on the proliferation of breast ER-positive cells and normal endothelial cells was not observed. Instead, a dose-dependent chemopreventive potential was demonstrated related to the inhibitory effect of extracts on migration and cell viability and by regulating MMP-9, N-cadherin, and E-cadherin gene expressions and VEGF secretion on all types of cells [77]. Aqueous and ethyl acetate extracts of red clover leaves showed protective effects by decreasing the activity of the xanthine oxidase enzyme in mice exposed to a hepatotoxic agent. The differences between antioxidant potential were mainly due to their phytochemical composition, with the ethyl acetate extract having a higher polyphenolic content higher than the aqueous one [28]. Recently, Akbaribazm M et al. have demonstrated that co-administration of *T. pratense* extract and doxorubicin reduced the side effects of the chemotherapeutic agent, inhibited the ROS generation, decreased the estradiol synthesis, and, overall, extended the survival rate of tumor-bearing mice [78].

The potential of *T. pratense* aerial part extracts to capture intracellular generated RNS has not been reported in the literature, yet a study showed that in noncellular context, a fraction enriched in red clover volatile oils has a greater potential to scavenge nitric oxide radicals than BHT and BHA, two very potent synthetic antioxidants [79]. Low concentrations of NO are sufficient to sustain its physiological functions, acting as an effector molecule in diverse biological processes. In contrast, supra-production of nitric oxide radical is associated with inflammatory conditions and carcinogenesis. Moreover, the cellular toxicity of NO increases greatly when it reacts with superoxide radicals, forming the highly reactive peroxynitrite anion that can rapidly initiate oxidative damage in cellular macromolecules [39]. Kolodziejczyk-Czepas J et al. reported that extracts and phenolic fractions of aerial parts of different species of *Trifolium* are capable, at noncytotoxic concentrations, of scavenging peroxynitrites generated in blood cells [80].

Regarding possible underlying mechanisms of cellular antioxidant properties of *T. pratense,* it can be attributed to the high content of flavonoids, especially kaempferol, quercetin, and rutin, followed by isoflavones. Additionally, cellular antioxidant effect can be exerted by phenolic acids such as caffeic, cinnamic, and chlorogenic acid, along with polyphenolic amides, namely, clovamides, which are all present in *T. pratense* extracts and have been tested separately in a cellular environment [80]. Therefore, they can act synergistically to directly scavenge free oxygen and nitrogen radicals in cells [28], yet indirect action of plant phenolics has been reported. The Nrf2- EpRE/ARE signaling pathway is the master regulator of the cellular oxidative stress system by controlling the activity of over 200 genes, which are mostly encoding Phase II enzymes involved in antioxidant defense and detoxification [12]. Lee et al. demonstrated that a red clover extract exerted a strong antioxidant activity on lipopolysaccharides-stimulated macrophages by decreasing the Nrf2 gene expression and phosphorylation of mitogen-activated protein kinases (MAPKs). Moreover, the extract acted as a potent anti-inflammatory agent by suppressing the nuclear translocation of NF-kB [27]. Individual isoflavones like biochanin A, which has a low radical scavenging activity, can effectively activate the Nrf2-ARE signaling pathways in cells with induced oxidative stress. One possible mechanism is the direct binding of biochanin A to Keap1, which is the cytoplasmatic suppressor of Nrf2, as molecular docking studies have demonstrated [81]. In addition, genistein can mimic the action of E2 in regulating antioxidant genes by binding to ERs and inducing signal transduction leading to phosphorylation of ERK1/2 kinase and, consequently, activating NFκB and increasing the expression of the antioxidant enzymes [6]. By their ability to interact with other nuclear receptors, like the aryl hydrocarbon receptor (AhR) and peroxisome proliferator-activated receptors (PPARs) that further influence metabolic processes, isoflavones present in *T. pratense* might contribute to activating the cellular oxidative defense system [82].

Although most of the beneficial effects of plant polyphenols can be ascribed to their antioxidant properties, emerging evidence indicates that they act as pro-oxidants and this might define their cytotoxic, antiproliferative, and apoptotic effects in pre-premalignant or malignant cells [11]. Various factors favor the transition of plant phenolics from antioxidant to pro-oxidant agents in the cellular environment [11]. The alkaline pH and a high concentration of polyphenolic compounds in the cell environment, along with the presence of oxygen and transition metal ions (particularly Fe^3+^ and Cu^2+^), can oxidize the phenolics to produce phenoxyl radicals and, finally, superoxide anions [11,83]. The superoxide in the mentioned conditions can be transformed further in hydrogen peroxide and, by metal ion-catalyzed reactions, can undergo into the most powerful oxidizing agent, such as a hydroxyl radical [11]. Despite having less scavenging potential compared with other flavonoids, both coumestrol and genistein can generate ROS in the presence of intracellular copper ions [83]. The oxidative stress leads to mitochondrial membrane depolarization and, finally, to inhibition of cellular proliferation and induced apoptosis in ER+ breast cancer cells [83]. Moreover, in the absence of transition metal ions, but in the presence of O_2_, plant phenolics can autoxidize, generating phenoxyl radicals and superoxide, thus increasing cellular oxidative stress [11].

In general, selective pro-oxidant cytotoxicity toward cancer cells has been reported for plant phenols, which has been explained mainly by the presence of more copper in malignant cells than in normal ones [83]. Moreover, as we mentioned before, cancer cells are characterized by a higher basal endogenous ROS level compared with precancerous and normal cells; therefore, the extra ROS generated by plant extracts will further sensitize cancer cells to induced oxidative stress [65].

In the present study, we further demonstrate that *T. pratense* extracts can act selectively on cell proliferation and viability depending on cells’ ER status and grade of malignancy. We observed a higher cytotoxic and antiproliferative effect, followed by cell apoptosis in highly invasive triple-negative cells (MDA-MB-468) incubated with both extracts. The dose- and time-dependent antiproliferative and apoptotic effect was a consequence of oxidative stress induced by *T. pratense* extracts.

Consistent with the results of cell viability, treatment with Tp-eth significantly inhibited the proliferation of MDA-MB-468 cells through the generation of ROS, as we demonstrated. The time-lapse microscopy allowed for the visualization of ROS overproduction in all three breast cell lines incubated with high-dose Tp-eth. The strong fluorescence signal appeared at 4 h after the extract was placed into cell media and it was maintained during the observed period of time. Next, the co-staining of ROS and mitochondria membrane revealed a significant reduction in mitochondria potential during 48 h of treatment. The ATP assessment confirmed that the ROS generation reduced the ATP levels as a consequence of mitochondria potential damage. The most affected were highly invasive triple-negative cells (MDA-MB-468), followed by adenocarcinoma ER-positive (MCF-7) and non-tumorigenic cells (MCF-12A), and the results were similar to what was obtained in cytotoxicity tests. The low dose of Tp-eth significantly affected ATP levels in highly invasive triple-negative cells, rather than in adenocarcinoma ER+ and normal breast cell lines. Furthermore, the evaluation of lipid peroxidation content as a marker of the oxidative stress process revealed the presence of high values of MDA in all tested cells incubated with high-dose Tp-eth.

Oxidative stress in cells occurs when the balance between ROS production and neutralization is disrupted due to the dysfunctional antioxidant defense system, which is formed by enzymes and endogenous antioxidant molecules, such as GSH [65]. The treatment with Tp-eth significantly reduced GSH levels in all cells; instead, the SOD activity was affected depending on the malignancy grade of cells. The GSH depletion, accompanied by reduced SOD activity and combined with increased lipid peroxidation implies that the high dose of Tp-eth extract disrupted the cellular antioxidant defense system and triggered oxidative stress-induced apoptosis. The morphological assessment of cells under 48 h of treatment with high-dose Tp-eth extract showed a decreasing percentage of viable cells depending on the grade of malignancy, with MDA-MB-468 cells more affected. Congruently the number of early and late apoptotic cells increased from non-tumorigenic to highly invasive cells. A small percentage of cells under secondary necrosis was observed, especially for triple-negative adenocarcinoma cells, as we expected. Cells incubated with low-dose of Tp-eth showed differential inhibition of the SOD activity depending on the tumorigenic grade and ER status, with MDA-MB-468 cells more affected versus MCF-7 cells. For normal breast cells, the smallest reduction in the SOD activity was noted, rather than GSH or ATP depletion, and an increase in lipid peroxidation.

No significant intracellular ROS generation was observed for high-dose Tp-aq extract, yet a slight depletion of ATP levels was noted for all types of cells. The least affected were MCF-12A cells (20%), followed by MCF-7 (30%), and the most decreasing level of ATP was observed for MDA-MB-468 cells (40%). However, even a moderate reduction in the ATP levels can unbalance the energetic status of cells and might further increase oxidative stress damage [7]. The possibility of Tp-aq, which contains less polyphenolics than Tp-eth, to disrupt the cellular antioxidant defense system cannot be ruled out. It is reasonable to assume that by extending the time of incubation with the aqueous extract, the oxidative stress process can be induced, as MDA and GSH level assessments show. A slightly, yet statistically relevant, increase in lipid peroxidation was observed for malignant cells, as well as a more consistent depletion of the GSH level compared with what was observed for non-tumorigenic cells. The SOD activity was affected more in invasive adenocarcinoma cells, MDA-MB-468 cells, confirming that the cells with a higher grade of tumorigenicity are more sensitive to plant phenolic pro-oxidant action.

Our results are consistent with what others reported. Dutta et al. demonstrated that soyabean and flaxseed aglycone-rich extracts can induce ROS generation and a decrease in SOD and glutathione peroxidase activities, thereby leading to accumulation of superoxide ions and peroxide in MCF-7 and MDA-MB-231 cells, depending on extract concentrations and ER status. The soy extract containing a high fraction of genistein and daidzein had the total quantity of isoflavones similar with what was found in *T. pratense* extracts used in our study. The apoptotic effect was more evident on triple-negative breast adenocarcinoma cells, and for MCF-7 cells, a decrease in proliferation rate due to cell cycle arrest was seen after 24 h incubation of cells with aglycone-rich extracts [84]. Furthermore, olive leaf and Annurca apple extracts at elevated concentrations caused a decrease in mitochondria functionality by generating ROS, consequently suppressing the activity of antioxidant enzymes and inducing a strong antiproliferative effect and apoptosis in breast and ovary cancer cells [36,85]. Moreover, the combination of a phenolic acid and a flavonoid (chlorogenic acid and cinnamaldehyde) selectively triggered breast cancer cell apoptosis and had little cytotoxic effect on normal breast cells. The decrease in mitochondrial membrane potential and reduced mitochondrial and glycolytic ATP production finally led to oxidative stress-induced apoptosis in breast malignant cells [86].

In general, phytoestrogens modulate the oxidative stress in breast cancer cells and breast tumors, which express both ERs depending on the ERα/ERβ ratio. For instance, activation of ERα promotes ROS production, while binding to ERβ inhibits the oxidative stress process. In MCF-7 cells, characterized by a high ERα/ERβ ratio, genistein treatment at high doses induced a decrease in antioxidant enzyme expression, namely, thioredoxin reductase and SOD, along with the upregulation of glutathione peroxidase expression. Thereby, the oxidative stress process was favored with consequent cell apoptosis and autophagy [87].

In cells lacking ER expression, genistein was able to induce cell death by mobilization of endogenous copper ions to produce ROS and enhance oxidative stress. The genistein treatment in MDA-MB-231 cells generated superoxide anions, which were rapidly converted to hydrogen peroxide. Then, in the presence of copper, the formation of hydroxyl radicals was possible, and after ROS accumulation, irreversible DNA damage, which led to cell death, was observed [87]. Furthermore, the dose inhibition of triple-negative cell growth by genistein was reported and was correlated with the reduction in NF-κB activity through Nocth-1 signaling pathway. Several genes expression was downregulated, namely, cyclin B1, Bcl-2, and Bcl-xL, which are related to cell cycle and programmed cell death [88]. In the normal breast cell line, genistein at physiological concentration can suppress the activities of cytochrome enzymes CYP1A1 and CYP1B1, consequently reducing the DNA damage induced by carcinogen agents, such as polycyclic aromatic hydrocarbons [87]. Thus, the isoflavones can have chemopreventive potential to block carcinogenesis.

Recently, Pons el al. demonstrated that genistein can modulate SIRT1 expression and, depending on ER ratio, can control ROS production, cell proliferation, and inflammatory response [89]. SIRT1 is a NAD⁺-dependent histone deacetylase that acts as major metabolic regulator. It reprograms inflammation through epigenetic mechanisms by altering histones and transcription factors such as NF-κB. In addition, SIRT1 regulates mitochondrial function and biogenesis, enhances antioxidant enzyme activities, and engages in complex signaling networks that modulate cellular responses to oxidative stress. This interplay is crucial for maintaining cellular health and longevity [90]. Recently, Oza MJ et al. have demonstrated that red clover extracts stimulated SIRT1 expression in pancreatic tissue of rats with type 2 diabetes, improved insulin sensitivity at the cellular level and lipid profile and promoted liver glycogen synthesis by modulating Akt-GSK signaling pathway [91].

The proteome profiling studies have confirmed that isoflavones can exert their biological activities through distinct molecular pathways depending on the cellular ER status and dose [92,93]. In breast adenocarcinoma ER + cells (MCF-7), low doses of isoflavones activated the signaling pathways related to sustained cell proliferation, such as tyrosine kinase signaling and cytoskeleton organization. In breast adenocarcinoma ER-, MDA-MB-231 cells, isoflavones altered lipid and phospholipid catabolism, extracellular matrix degradation, mRNA splicing, and, thus, inhibited cell proliferation. Furthermore, in MCF-7 cells depending on isoflavone concentrations, different changes in the proteome have been observed, mainly related to cell cycle alterations [93].

Recently, flavonoids have been reported to exert biphasic dose response on cellular oxidative stress and proliferation process in breast cancer cells. Low doses of flavonoids such as quercetin and apigenin promoted breast human and murine cancer cell growth independent of ER status or degree of malignancy. Moreover, they exerted antioxidant activity, alleviated the MDA content, and restored the SOD activity in LPS-induced oxidative stress in MCF-7 cells. At high doses, flavonoids triggered apoptosis via oxidative stress, which was evidenced by ROS generation, higher MDA levels, and downregulation of the SOD activity [10]. However, in recent years, several in vitro and in vivo studies have identified the biphasic dose response as a common feature of herbal extracts rich in bioactive compounds with antioxidant, anti-inflammatory, and estrogenic properties [94,95,96].

## 5. Conclusions and Future Perspective

Given the rapid increase in global consumption of plant-based supplements and the fact that phytochemicals with estrogenic and antioxidant properties are commonly present in the human daily diet, in vitro studies investigating their dual biological efficacy and establishing the threshold of toxicological concern are still necessary. The red clover extracts analyzed in this study have a moderate content of isoflavones and phytochemicals with antioxidant properties. The study demonstrated that *T. pratense* extracts exerted a biphasic dose response on the proliferation and oxidative stress of breast cells depending on their grade of malignancy and ER levels of expression. For cells being either non-tumorigenic or having a moderate grade of malignancy, at low doses, a stimulatory effect on cell proliferation and strong cellular antioxidant activity was established. The cellular proliferation effect induced in ER+ breast adenocarcinoma cells was ER-dependent. Furthermore, both *T. pratense* extracts showed an estrogenic effect, which was higher than E2 alone. The proliferation of non-tumorigenic breast cells was induced by an ER-independent mechanism, so the possibility of extracts to act as epigenetic modulators could be a future perspective of the study.

A distinct monotonic cytotoxic effect depending on time and extract concentration was observed in the adenocarcinoma highly invasive ER- cell line, which is reflected by a decrease in proliferation and oxidative stress-induced apoptosis, more strongly evidenced by *T. pratense* ethanolic extract treatment.

Actually, one of the main results obtained in this study is that high-dose *T. pratense* ethanolic extracts can generate intracellular ROS, leading to mitochondrial dysfunction, ATP and GSH level depletion, an increase in lipid peroxidation, and a reduction in SOD activity in all tested cells. The kinetics of the ROS-induced apoptosis process should be studied in the future to understand the underlying molecular mechanism of action of ethanolic extracts. The loss of mitochondrial membrane integrity and GSH depletion earlier than modulated expression of Bcl-2 family proteins might suggest that plant extracts can directly target mitochondria [97]. Moreover, the subsequent cytochrome c release, followed by caspase-9 activation and cleavage of caspase-3, could link the oxidative stress induced by ethanolic extract to its pro-apoptotic effect [98].

However, a dependency of polyphenolic and flavonoid content was observed as *T. pratense* aqueous extract, which has only half of the phytochemical content, showed rather moderate modulation of oxidative markers. Nevertheless, the intensity of oxidative stress induced by *T. pratense* extracts, followed by apoptosis, was stronger in highly invasive adenocarcinoma cells. Additionally, this is the first study showing that *T. pratense* aqueous extract at low doses has a protective role in normal breast cells lacking ER expression, by enhancing cell proliferation and inducing a noncytotoxic, protective effect. Moreover, both extracts at high doses showed selective toxicity toward tumor cells, with the proliferation rate and oxidative stress markers being less affected in normal cells.

Thus, our results confirmed that plant extracts rich in phytoestrogens and phytochemicals with radical scavenging and ion-reducing capacities can induce a biphasic dose response on breast cell proliferation and oxidative stress processes, depending on ER status and grade of malignancy.

Knowledge of the biological properties and phytochemical content of herbs is required prior to further exploring them in drug development, the food industry, and, also, for chemoprevention and as an adjuvant therapeutic approach in cancer.

Although results from in vitro studies are not always predictive of the chemopreventive potential of herbs in humans, they constitute a valuable tool for studying the biological effects on cellular processes and molecular targets involved in carcinogenesis and tumor growth. More studies are clearly needed to explore the phytochemical and plant extract hormetic biphasic dose response in order to understand the adaptive cellular response for further exploitation in pharmacology and medicine.

Nevertheless, future in vivo and clinical studies are necessary to confirm that *T. pratense* extracts are effective in preventing symptoms associated with estrogen deficiency during menopause or other associated diseases. It will be necessary to take into account all the factors that may impact digestion, absorption, bioavailability, and, ultimately, the efficacy of the phytocomponents of plant extracts. Evidently, it is essential to assess the biological effects of *T. pratense* at multiple levels in order to differentiate its impact on various human physiological or pathological states, including hormone-dependent premalignancies.

## Figures and Tables

**Table 1:** TPC and TFC of *T. pratense* extracts.

*T. pratense* Extract	TPC (mg GAE/g)	TFC (mg QE/g)
Tp-aq	6.49 ± 0.17 *	2.99 ± 0.15 *
Tp-eth	9.66 ± 0.18 *	6.05 ± 0.16 *

* concentrations are expressed as mean ± SEM.

**Table 2:** The isoflavones content of T. pratense extracts detected by the HPLC-UV method.

Sample	Daidzein	Genistein	Formononetin	Biochanin A	Total Isoflavones
Tp-aq	41.37 ± 2.37 *	62.58 ± 3.05 *	369.46 ± 5.24 *	130.53 ± 3.04 *	603.89 ± 13.7 *
Tp-eth	40.92 ± 0.94 *	160.15 ± 4.32 *	139.76 ± 3.81 *	295.60 ± 7.23 *	636.43 ± 16.3 *

* μg/g extract. All concentrations are expressed as mean ± SEM.

**Table 3:** Antioxidant capacity of *T. pratense* extracts.

*T. pratense* Extract	DPPH (mg BHT/g)	FRAP (mg Fe^2+^/g)	CUPRAC (mg Trolox/g)
Tp-aq	2.02 ± 0.05 *	1.96 ± 0.08 *	3.89 ± 0.09 *
Tp-eth	4.61 ± 0.12 *	2.26 ± 0.11 *	6.98 ± 0.29 *

* concentrations are expressed as mean ± SEM.

## Data Availability

Data is contained within the article and Supplementary Materials.

## Supplementary Materials

The following supporting information can be downloaded at: https://www.mdpi.com/article/10.3390/antiox13121435/s1, Figure S1: Intracellular ROS generation by Tp-eth at a concentration of 1.0 mg/mL.

## References

[1] Djaoudene O., Romano A., Bradai Y.D., Zebiri F., Ouchene A., Yousfi Y., Amrane-Abider M., Sahraoui-Remini Y., Madani K. (2023). A Global Overview of Dietary Supplements: Regulation, Market Trends, Usage during the COVID-19 Pandemic, and Health Effects. Nutrients.

[2] Pan S.-Y., Zhou S.-F., Gao S.-H., Yu Z.-L., Zhang S.-F., Tang M.-K., Sun J.-N., Ma D.-L., Han Y.-F., Fong W.-F. (2013). New Perspectives on How to Discover Drugs from Herbal Medicines: CAM’s Outstanding Contribution to Modern Therapeutics. Evid.-Based Complement. Altern. Med..

[3] George B.P., Chandran R., Abrahamse H. (2021). Role of Phytochemicals in Cancer Chemoprevention: Insights. Antioxidants.

[4] Veiga M., Costa E.M., Silva S., Pintado M. (2018). Impact of plant extracts upon human health: A review. Crit. Rev. Food Sci. Nutr..

[5] Pop S., Enciu A.M., Tarcomnicu I., Gille E., Tanase C. (2019). Phytochemicals in cancer prevention: Modulating epigenetic alterations of DNA methylation. Phytochem. Rev..

[6] Ionescu V.S., Popa A., Alexandru A., Manole E., Neagu M., Pop S. (2021). Dietary Phytoestrogens and Their Metabolites as Epigenetic Modulators with Impact on Human Health. Antioxidants.

[7] Wang Y., Qi H., Liu Y., Duan C., Liu X., Xia T., Chen D., Piao H.-L., Liu H.-X. (2021). The double-edged roles of ROS in cancer prevention and therapy. Theranostics.

[8] Speisky H., Shahidi F., Costa de Camargo A., Fuentes J. (2022). Revisiting the Oxidation of Flavonoids: Loss, Conservation or Enhancement of Their Antioxidant Properties. Antioxidants.

[9] Wendt J., Knudsen B., Frame L.A. (2024). Are Supra-Physiological Plant-Based Antioxidants Ready for the Clinic? A Scoping Review of Hormetic Influences Driving Positive Clinical Outcomes. Glob. Adv. Integr. Med. Health.

[10] Xi X., Wang J., Qin Y., You Y., Huang W., Zhan J. (2022). The Biphasic Effect of Flavonoids on Oxidative Stress and Cell Proliferation in Breast Cancer Cells. Antioxidants.

[11] León-González A.J., Auger C., Schini-Kerth V.B. (2015). Pro-oxidant activity of polyphenols and its implication on cancer chemoprevention and chemotherapy. Biochem. Pharmacol..

[12] Russo G.L., Spagnuolo C., Russo M. (2024). Reassessing the role of phytochemicals in cancer chemoprevention. Biochem. Pharmacol..

[13] Lecomte S., Demay F., Ferrière F., Pakdel F. (2017). Phytochemicals Targeting Estrogen Receptors: Beneficial Rather Than Adverse Effects?. Int. J. Mol. Sci..

[14] Jodynis-Liebert J., Kujawska M. (2020). Biphasic Dose-Response Induced by Phytochemicals: Experimental Evidence. J. Clin. Med..

[15] Canivenc-Lavier M.-C., Bennetau-Pelissero C. (2023). Phytoestrogens and Health Effects. Nutrients.

[16] Calabrese E.J. (2021). Hormesis Mediates Acquired Resilience: Using Plant-Derived Chemicals to Enhance Health. Annu. Rev. Food Sci. Technol..

[17] Barreiro-Sisto U., Fernández-Fariña S., González-Noya A.M., Pedrido R., Maneiro M. (2024). Enemies or Allies? Hormetic and Apparent Non-Dose-Dependent Effects of Natural Bioactive Antioxidants in the Treatment of Inflammation. Int. J. Mol. Sci..

[18] Wan Y., Liu J., Mai Y., Hong Y., Jia Z., Tian G., Liu Y., Liang H., Liu J. (2024). Current advances and future trends of hormesis in disease. NPJ Aging.

[19] Kolodziejczyk-Czepas J. (2016). Trifoliumspecies—The latest findings on chemical profile, ethnomedicinal use and pharmacological properties. J. Pharm. Pharmacol..

[20] Almeida I.M.C., Rodrigues F., Sarmento B., Alves R.C., Oliveira M.B.P.P. (2015). Isoflavones in food supplements: Chemical profile, label accordance and permeability study in Caco-2 cells. Food Funct..

[21] Myers S.P., Vigar V. (2017). Effects of a standardised extract of *Trifolium pratense* (Promensil) at a dosage of 80mg in the treatment of menopausal hot flushes: A systematic review and meta-analysis. Phytomedicine.

[22] Luís Â., Domingues F., Pereira L. (2018). Effects of red clover on perimenopausal and postmenopausal women’s blood lipid profile: A meta-analysis. Climacteric.

[23] EFSA Panel on Food Additives and Nutrient Sources added to Food (ANS) (2015). Risk assessment for peri- and post-menopausal women taking food supplements containing isolated isoflavones. EFSA J..

[24] Khazaei M., Pazhouhi M., Khazaei S. (2018). Evaluation of Hydro-Alcoholic Extract of *Trifolium pratens* L. for Its Anti-Cancer Potential on U87MG Cell Line. Cell J..

[25] Akbaribazm M., Khazaei M.R., Khazaei M. (2020). Phytochemicals and antioxidant activity of alcoholic/hydroalcoholic extract of *Trifolium pratense*. Chin. Herb. Med..

[26] Albulescu L., Bercu V., Manole E., Suciu A., Luntraru C., Neagu M., Pop S. In vitro studies of antioxidant and estrogenic activities of *Trifolium pratense* L. extracts on human adenocarcinoma and non-tumorigenic breast cell lines. Proceedings of the GA—69th Annual Meeting 2021.

[27] Lee S.A., Park B.-R., Moon S.-M., Han S.H., Kim C.S. (2018). Anti-inflammatory potential of *Trifolium pratense* L. leaf extract in LPS-stimulated RAW264.7 cells and in a rat model of carrageenan-induced inflammation. Arch. Physiol. Biochem..

[28] Kaurinovic B., Popovic M., Vlaisavljevic S., Schwartsova H., Vojinovic-Miloradov M. (2012). Antioxidant Profile of *Trifolium pratense* L.. Molecules.

[29] Dunlap T.L., Howell C.E., Mukand N., Chen S.-N., Pauli G.F., Dietz B.M., Bolton J.L. (2017). Red Clover Aryl Hydrocarbon Receptor (AhR) and Estrogen Receptor (ER) Agonists Enhance Genotoxic Estrogen Metabolism. Chem. Res. Toxicol..

[30] Burdette J.E., Liu J., Lantvit D., Lim E., Booth N., Bhat K.P., Hedayat S., Van Breemen R.B., Constantinou A.I., Pezzuto J.M. (2002). *Trifolium pratense* (red clover) exhibits estrogenic effects in vivo in ovariectomized Sprague-Dawley rats. J. Nutr..

[31] Saviranta N.M.M., Julkunen-Tiitto R., Oksanen E., Karjalainen R.O. (2010). Leaf phenolic compounds in red clover (*Trifolium pratense* L.) induced by exposure to moderately elevated ozone. Environ. Pollut..

[32] Chandra S., Khan S., Avula B., Lata H., Yang M.H., ElSohly M.A., Khan I.A., Ohta Y. (2014). Assessment of Total Phenolic and Flavonoid Content, Antioxidant Properties, and Yield of Aeroponically and Conventionally Grown Leafy Vegetables and Fruit Crops: A Comparative Study. Evid.-Based Complement. Altern. Med..

[33] Anil Kumar M., Vinukonda V.P., Giri A. (2016). A Validated RP-HPLC Method for Simultaneous Determination of Daidzein, Genistein, Formononetin and Biochanin A from In Vitro Cultured Cells of *Trifolium pratense* L. (Fabaceae). Pharma Chem..

[34] Munteanu I.G., Apetrei C. (2021). Analytical Methods Used in Determining Antioxidant Activity: A Review. Int. J. Mol. Sci..

[35] Szydłowska-Czerniak A., Tułodziecka A., Szłyk E. (2011). Determination of Antioxidant Capacity of Unprocessed and Processed Food Products by Spectrophotometric Methods. Food Anal. Methods.

[36] Benot-Dominguez R., Tupone M.G., Castelli V., d’Angelo M., Benedetti E., Quintiliani M., Cinque B., Forte I.M., Cifone M.G., Ippoliti R. (2021). Olive leaf extract impairs mitochondria by pro-oxidant activity in MDA-MB-231 and OVCAR-3 cancer cells. Biomed. Pharmacother..

[37] Ke N., Wang X., Xu X., Abassi Y.A. (2011). The xCELLigence System for Real-Time and Label-Free Monitoring of Cell Viability. Mammalian Cell Viability.

[38] Soto A.M., Sonnenschein C., Chung K.L., Fernandez M.F., Olea N., Serrano F.O. (1995). The E-SCREEN assay as a tool to identify estrogens: An update on estrogenic environmental pollutants. Environ. Health Perspect..

[39] Feelisch M. (1998). The use of nitric oxide donors in pharmacological studies. Naunyn-Schmiedeberg’s Arch. Pharmacol..

[40] Vanden Berghe T., Grootjans S., Goossens V., Dondelinger Y., Krysko D.V., Takahashi N., Vandenabeele P. (2013). Determination of apoptotic and necrotic cell death in vitro and in vivo. Methods.

[41] Gligor O., Clichici S., Moldovan R., Muntean D., Vlase A.-M., Nadăș G.C., Novac C.Ș., Filip G.A., Vlase L., Crișan G. (2022). Red Clover and the Importance of Extraction Processes—Ways in Which Extraction Techniques and Parameters Affect *Trifolium pratense* L. Extracts’ Phytochemical Profile and Biological Activities. Processes.

[42] Antonescu I.-A. (2019). Comparative Phytochemical and Antioxidative Characterization of *Trifolium pratense* L. and *Ocimum basilicum* L.. Farmacia.

[43] Gościniak A., Szulc P., Zielewicz W., Walkowiak J., Cielecka-Piontek J. (2023). Multidirectional Effects of Red Clover (*Trifolium pratense* L.) in Support of Menopause Therapy. Molecules.

[44] Schmitz G.E., Sullivan M.L., Hatfield R.D. (2007). Three Polyphenol Oxidases from Red Clover (*Trifolium pratense*) Differ in Enzymatic Activities and Activation Properties. J. Agric. Food Chem..

[45] Vlaisavljević S., Kaurinović B., Popović M., Vasiljević S. (2017). Profile of phenolic compounds in *Trifolium pratense* L. extracts at different growth stages and their biological activities. Int. J. Food Prop..

[46] Kazlauskaite J.A., Ivanauskas L., Marksa M., Bernatoniene J. (2022). The Effect of Traditional and Cyclodextrin-Assisted Extraction Methods on *Trifolium pratense* L. (Red Clover) Extracts Antioxidant Potential. Antioxidants.

[47] Gouda M., El-Din Bekhit A., Tang Y., Huang Y., Huang L., He Y., Li X. (2021). Recent innovations of ultrasound green technology in herbal phytochemistry: A review. Ultrason. Sonochem..

[48] Kazlauskaite J.A., Ivanauskas L., Bernatoniene J. (2021). Novel Extraction Method Using Excipients to Enhance Yield of Genistein and Daidzein in *Trifolium pratensis* L.. Pharmaceutics.

[49] Chiang W.-D., Shih C.-J., Chu Y.-H. (2001). Optimization of acid hydrolysis conditions for total isoflavones analysis in soybean hypocotyls by using RSM. Food Chem..

[50] Temerdashev Z.A., Chubukina T.K., Vinitskaya E.A., Nagalevskii M.V., Kiseleva N.V. (2021). Assessment of the Concentrations of Isoflavonoids in Red Clover (*Trifolium pratense* L.) of the Fabaceae Family Using Extraction by Different Methods. J. Anal. Chem..

[51] Hanganu D., Vlase L., Olah N. (2010). LC/MS analysis of isoflavones from Fabaceae species extracts. Farmacia.

[52] Mikulić M., Atanacković Krstonošić M., Kladar N., Vasiljević S., Katanski S., Mamlić Z., Rakić D., Cvejić J. (2023). Phytochemical Composition of Different Red Clover Genotypes Based on Plant Part and Genetic Traits. Foods.

[53] Dai J., Mumper R.J. (2010). Plant Phenolics: Extraction, Analysis and Their Antioxidant and Anticancer Properties. Molecules.

[54] Kukavica B., Škondrić S., Trifković T., Mišić D., Gašić U., Topalić-Trivunović L., Savić A., Velemir A., Davidović-Plavšić B., Šešić M. (2024). Comparative polyphenolic profiling of five ethnomedicinal plants and their applicative potential in the treatment of type 2 diabetes. J. Ethnopharmacol..

[55] Jakubczyk K., Łukomska A., Gutowska I., Kochman J., Janił J., Janda K. (2021). Edible Flowers Extracts as a Source of Bioactive Compounds with Antioxidant Properties—In Vitro Studies. Appl. Sci..

[56] Tundis R., Marrelli M., Conforti F., Tenuta M., Bonesi M., Menichini F., Loizzo M. (2015). *Trifolium pratense* and *T. repens* (Leguminosae): Edible Flower Extracts as Functional Ingredients. Foods.

[57] Verpoorte R., Contin A., Memelink J. (2002). Biotechnology for the production of plant secondary metabolites. Phytochem. Rev..

[58] Reis A., Scopel M., Zuanazzi J.A.S. (2018). *Trifolium pratense*: Friable calli, cell culture protocol and isoflavones content in wild plants, in vitro and cell cultures analyzed by UPLC. Rev. Bras. Farmacogn..

[59] Calderón-Montaño J.M., Martínez-Sánchez S.M., Jiménez-González V., Burgos-Morón E., Guillén-Mancina E., Jiménez-Alonso J.J., Díaz-Ortega P., García F., Aparicio A., López-Lázaro M. (2021). Screening for Selective Anticancer Activity of 65 Extracts of Plants Collected in Western Andalusia, Spain. Plants.

[60] Fu H., Fu W., Sun M., Shou Q., Zhai Y., Cheng H., Teng L., Mou X., Li Y., Wan S. (2011). Kinetic Cellular Phenotypic Profiling: Prediction, Identification, and Analysis of Bioactive Natural Products. Anal. Chem..

[61] Sweeney M.F., Sonnenschein C., Soto A.M. (2018). Characterization of MCF-12A cell phenotype, response to estrogens, and growth in 3D. Cancer Cell Int..

[62] Spagnuolo P., Rasini E., Luini A., Legnaro M., Luzzani M., Casareto E., Carreri M., Paracchini S., Marino F., Cosentino M. (2014). Isoflavone content and estrogenic activity of different batches of red clover (*Trifolium pratense* L.) extracts: An in vitro study in MCF-7 cells. Fitoterapia.

[63] Zhao H., Wu L., Yan G., Chen Y., Zhou M., Wu Y., Li Y. (2021). Inflammation and tumor progression: Signaling pathways and targeted intervention. Signal Transduct. Target. Ther..

[64] Dzah C.S., Zhang H., Gobe V., Asante-Donyinah D., Duan Y. (2024). Anti- and pro-oxidant properties of polyphenols and their role in modulating glutathione synthesis, activity and cellular redox potential: Potential synergies for disease management. Adv. Redox Res..

[65] Sarmiento-Salinas F.L., Delgado-Magallón A., Montes-Alvarado J.B., Ramírez-Ramírez D., Flores-Alonso J.C., Cortés-Hernández P., Reyes-Leyva J., Herrera-Camacho I., Anaya-Ruiz M., Pelayo R. (2019). Breast Cancer Subtypes Present a Differential Production of Reactive Oxygen Species (ROS) and Susceptibility to Antioxidant Treatment. Front. Oncol..

[66] Couto N., Wood J., Barber J. (2016). The role of glutathione reductase and related enzymes on cellular redox homoeostasis network. Free. Radic. Biol. Med..

[67] Basu P., Maier C. (2018). Phytoestrogens and breast cancer: In vitro anticancer activities of isoflavones, lignans, coumestans, stilbenes and their analogs and derivatives. Biomed. Pharmacother..

[68] Pop S., Enciu A.M., Necula L.G., Tanase C. (2018). Long non-coding RNAs in brain tumours: Focus on recent epigenetic findings in glioma. J. Cell. Mol. Med..

[69] Xie Q., Bai Q., Zou L.Y., Zhang Q.Y., Zhou Y., Chang H., Yi L., Zhu J.D., Mi M.T. (2014). Genistein inhibits DNA methylation and increases expression of tumor suppressor genes in human breast cancer cells. Genes Chromosomes Cancer.

[70] Wang L., Li L., Han Q., Wang X., Zhao D., Liu J. (2020). Identification and biological evaluation of natural product Biochanin A. Bioorg. Chem..

[71] Oza M.J., Kulkarni Y.A. (2019). Formononetin attenuates kidney damage in type 2 diabetic rats. Life Sci..

[72] Overk C.R., Yao P., Chadwick L.R., Nikolic D., Sun Y., Cuendet M.A., Deng Y., Hedayat A.S., Pauli G.F., Farnsworth N.R. (2005). Comparison of the in Vitro Estrogenic Activities of Compounds from Hops (*Humulus lupulus*) and Red Clover (*Trifolium pratense*). J. Agric. Food Chem..

[73] Chen F.P., Chien M.H. (2014). Phytoestrogens induce differential effects on both normal and malignant human breast cellsin vitro. Climacteric.

[74] Moon Y.J., Brazeau D.A., Morris M.E. (2007). Effects of Flavonoids Genistein and Biochanin A on Gene Expression and Their Metabolism in Human Mammary Cells. Nutr. Cancer.

[75] Beetch M., Lubecka K., Kristofzski H., Suderman M., Stefanska B. (2018). Subtle Alterations in DNA Methylation Patterns in Normal Cells in Response to Dietary Stilbenoids. Mol. Nutr. Food Res..

[76] Gligor O., Clichici S., Moldovan R., Decea N., Vlase A.-M., Fizeșan I., Pop A., Virag P., Filip G.A., Vlase L. (2023). An In Vitro and In Vivo Assessment of Antitumor Activity of Extracts Derived from Three Well-Known Plant Species. Plants.

[77] Zakłos-Szyda M., Budryn G. (2020). The Effects of *Trifolium pratense* L. Sprouts’ Phenolic Compounds on Cell Growth and Migration of MDA-MB-231, MCF-7 and HUVEC Cells. Nutrients.

[78] Akbaribazm M., Khazaei M.R., Khazaei M. (2020). *Trifolium pratense* L. (red clover) extract and doxorubicin synergistically inhibits proliferation of 4T1 breast cancer in tumor-bearing BALB/c mice through modulation of apoptosis and increase antioxidant and anti-inflammatory related pathways. Food Sci. Nutr..

[79] Vlaisavljevic S., Kaurinovic B., Popovic M., Djurendic-Brenesel M., Vasiljevic B., Cvetkovic D., Vasiljevic S. (2014). *Trifolium pratense* L. as a Potential Natural Antioxidant. Molecules.

[80] Kolodziejczyk-Czepas J., Nowak P., Moniuszko-Szajwaj B., Kowalska I., Stochmal A. (2015). Free radical scavenging actions of threeTrifoliumspecies in the protection of blood plasma antioxidant capacityin vitro. Pharm. Biol..

[81] Liang F., Cao W., Huang Y., Fang Y., Cheng Y., Pan S., Xu X. (2019). Isoflavone biochanin A, a novel nuclear factor erythroid 2-related factor 2 (Nrf2)-antioxidant response element activator, protects against oxidative damage in HepG2 cells. BioFactors.

[82] Chavda V.P., Chaudhari A.Z., Balar P.C., Gholap A., Vora L.K. (2024). Phytoestrogens: Chemistry, potential health benefits, and their medicinal importance. Phytother. Res..

[83] Zafar A., Singh S., Naseem I. (2017). Cytotoxic activity of soy phytoestrogen coumestrol against human breast cancer MCF-7 cells: Insights into the molecular mechanism. Food Chem. Toxicol..

[84] Dutta S., Khanna A. (2016). Aglycone rich extracts of phytoestrogens cause ROS-mediated DNA damage in breast carcinoma cells. Biomed. Pharmacother..

[85] D’Angelo S., Martino E., Ilisso C.P., Bagarolo M.L., Porcelli M., Cacciapuoti G. (2017). Pro-oxidant and pro-apoptotic activity of polyphenol extract from Annurca apple and its underlying mechanisms in human breast cancer cells. Int. J. Oncol..

[86] Schuster C., Wolpert N., Moustaid-Moussa N., Gollahon L.S. (2022). Combinatorial Effects of the Natural Products Arctigenin, Chlorogenic Acid, and Cinnamaldehyde Commit Oxidation Assassination on Breast Cancer Cells. Antioxidants.

[87] Uifălean A., Schneider S., Ionescu C., Lalk M., Iuga C. (2015). Soy Isoflavones and Breast Cancer Cell Lines: Molecular Mechanisms and Future Perspectives. Molecules.

[88] Pan H., Zhou W., He W.E.I., Liu X., Ding Q., Ling L., Zha X., Wang S. (2012). Genistein inhibits MDA-MB-231 triple-negative breast cancer cell growth by inhibiting NF-κB activity via the Notch-1 pathway. Int. J. Mol. Med..

[89] Pons D.G., Vilanova-Llompart J., Gaya-Bover A., Alorda-Clara M., Oliver J., Roca P., Sastre-Serra J. (2019). The phytoestrogen genistein affects inflammatory-related genes expression depending on the ERα/ERβ ratio in breast cancer cells. Int. J. Food Sci. Nutr..

[90] Singh C.K., Chhabra G., Ndiaye M.A., Garcia-Peterson L.M., Mack N.J., Ahmad N. (2018). The Role of Sirtuins in Antioxidant and Redox Signaling. Antioxid. Redox Signal..

[91] Oza M.J., Kulkarni Y.A. (2020). *Trifolium pratense* (Red Clover) Improve SIRT1 Expression and Glycogen Content in High Fat Diet-Streptozotocin Induced Type 2 Diabetes in Rats. Chem. Biodivers..

[92] Lavigne J.A., Takahashi Y., Chandramouli G.V.R., Liu H., Perkins S.N., Hursting S.D., Wang T.T.Y. (2007). Concentration-dependent effects of genistein on global gene expression in MCF-7 breast cancer cells: An oligo microarray study. Breast Cancer Res. Treat..

[93] Ilieș M., Uifălean A., Pașca S., Dhople V.M., Lalk M., Iuga C.A., Hammer E. (2020). From Proteomics to Personalized Medicine: The Importance of Isoflavone Dose and Estrogen Receptor Status in Breast Cancer Cells. J. Pers. Med..

[94] Macrì R., Musolino V., Gliozzi M., Carresi C., Maiuolo J., Nucera S., Scicchitano M., Bosco F., Scarano F., Ruga S. (2020). *Ferula* L. Plant Extracts and Dose-Dependent Activity of Natural Sesquiterpene Ferutinin: From Antioxidant Potential to Cytotoxic Effects. Molecules.

[95] Calabrese E.J., Dhawan G., Kapoor R., Agathokleous E., Calabrese V. (2023). Moringa induces its beneficial effect via hormesis. Nutrition Research Reviews.

[96] Basu P., Meza E., Bergel M., Maier C. (2019). Estrogenic, Antiestrogenic and Antiproliferative Activities of Euphorbia bicolor (Euphorbiaceae) Latex Extracts and Its Phytochemicals. Nutrients.

[97] Huang Y.T., Huang Y.H., Hour T.C., Pan B.S., Liu Y.C., Pan M.H. (2006). Apoptosis-inducing active components from *Corbicula fluminea* through activation of caspase-2 and production of reactive oxygen species in human leukemia HL-60 cells. Food Chem. Toxicol..

[98] Chen W.-J., Huang Y.-T., Wu M.-L., Huang T.-C., Ho C.-T., Pan M.-H. (2008). Induction of apoptosis by vitamin D2, ergocalciferol, via reactive oxygen species generation, glutathione depletion, and caspase activation in human leukemia Cells. J. Agric. Food Chem..

