# Supplementary material for: Differential Biological Effects of Trifolium pratense Extracts—In Vitro Studies on Breast Cancer Models
Source: Antioxidants (Basel). 2024 Nov 22;13(12):1435. doi: 10.3390/antiox13121435 (PMC11672829; doi:10.3390/antiox13121435)
Supplement: Supplementary file 1 [file antioxidants-13-01435-s001.zip › antioxidants-3285724-supplementary.pdf]

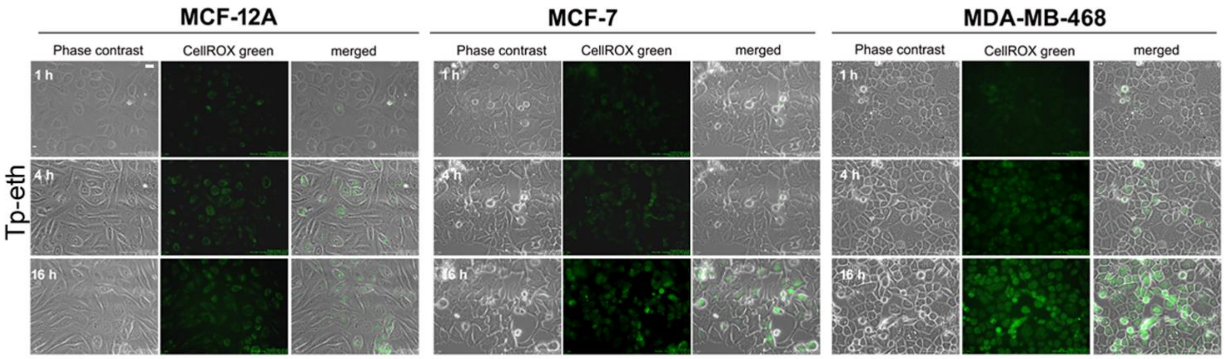

Figure S1. Intracellular ROS generation by Tp-eth at concentration of 1.0 mg/ml. The cells previously loaded with CellROX were treated with 1.0 mg/mL concentration of Tp-eth extract and observed under the microscope for 24 hours. The representative images obtained at 1, 4 and 16 hours after starting the incubation of MCF-12A, MCF-7 and MDA-MB-468 cells with Tp-eth extract are presented. The live-cells imaging was performed using a BioStation IM platform (Nikon); the time-lapse images were taken with 20x objective on phase contrast and green channel. Scale bar = 10 μm.
